# Short- and long-term polystyrene nano- and microplastic exposure promotes oxidative stress and divergently affects skin cell architecture and Wnt/beta-catenin signaling

**DOI:** 10.1186/s12989-023-00513-1

**Published:** 2023-01-16

**Authors:** Anke Schmidt, Walison Augusto da Silva Brito, Debora Singer, Melissa Mühl, Julia Berner, Fariba Saadati, Christina Wolff, Lea Miebach, Kristian Wende, Sander Bekeschus

**Affiliations:** 1 ZIK plasmatis, Leibniz Institute for Plasma Science and Technology (INP), Felix-Hausdorff-Str. 2, 17489 Greifswald, Germany; 2 Department of General Pathology, State University of Londrina, Rodovia Celso Garcia Cid, Londrina, Brazil; 3 Department Oral, Maxillofacial, and Plastic Surgery, Greifswald University Medical Center, Ferdinand-Sauerbruch-Str., Greifswald, Germany; 4 Department of General, Visceral, Thoracic, and Vascular Surgery, Greifswald University Medical Center, Ferdinand-Sauerbruch-Str., Greifswald, Germany

**Keywords:** Environment, Inflammation, Nrf2, Plastic particles, Toxicity, Uptake

## Abstract

Nano- and microplastic particles (NMP) are strong environmental contaminants affecting marine ecosystems and human health. The negligible use of biodegradable plastics and the lack of knowledge about plastic uptake, accumulation, and functional consequences led us to investigate the short- and long-term effects in freshly isolated skin cells from mice. Using fluorescent NMP of several sizes (200 nm to 6 µm), efficient cellular uptake was observed, causing, however, only minor acute toxicity as metabolic activity and apoptosis data suggested, albeit changes in intracellular reactive species and thiol levels were observed. The internalized NMP induced an altered expression of various targets of the nuclear factor-2-related transcription factor 2 pathway and were accompanied by changed antioxidant and oxidative stress signaling responses, as suggested by altered heme oxygenase 1 and glutathione peroxide 2 levels. A highly increased beta-catenin expression under acute but not chronic NMP exposure was concomitant with a strong translocation from membrane to the nucleus and subsequent transcription activation of Wnt signaling target genes after both single-dose and chronic long-term NMP exposure. Moreover, fibroblast-to-myofibroblast transdifferentiation accompanied by an increase of α smooth muscle actin and collagen expression was observed. Together with several NMP-induced changes in junctional and adherence protein expression, our study for the first time elucidates the acute and chronic effects of NMP of different sizes in primary skin cells' signaling and functional biology, contributing to a better understanding of nano- and microplastic to health risks in higher vertebrates.

**Supplementary Information:**

The online version contains supplementary material available at 10.1186/s12989-023-00513-1.

## Introduction

Plastic and mixtures of different plastic types are assumed to be contaminants in environmental sources such as water [1, 2]. Plastic contaminants are polymeric, *e.g.*, polystyrene, polyethylene, polyurethane, polypropylene, and polyvinyl chloride [3]. In vertebrates, including humans, the uptake, accumulation, and distribution of nano- and microplastic particles (NMP) in cells and tissues and their biomedical relevance are still largely unexplored [4]. The mechanisms of disruption, penetration, adsorption, and endocytosis are currently being discussed as possible ways of interaction and entry of NMP into cells and tissue [5] with several toxic consequences [6, 7]. In the past years, the oral uptake of NMP and absorption via the gastrointestinal tract have been the focus of research [8, 9]. The small insoluble NMP up to 10 µm can penetrate all organs [10]. Moreover, the response to chronic, long-term NMP exposure is probably more substantial to the accumulation observed in our environment. The continuous penetration of NMP through the skin via cleaners and foams could have toxic effects leading to an accumulation in the digestive tract and liver [11]. Preliminary evidence of long-term damage caused by NMP was found in rats' reproductive organs [12]. In murine models, it has been found that exposure to NMP alters the microbiome in the intestine [13], affects liver fat metabolism [14, 15], and influences host–pathogen interaction [16]. Additionally, the addition of small NMP provokes metabolic dysfunctions in first- and second-generation offspring [17, 18], regardless of sex [19], affecting the uterus [20] and milk production [21]. Moreover, several routes of NMP exposure, including oral intake via drinking water, marine or other foods, and inhalation, could adversely affect human health [22]. Thus, NMP can be inhaled through the air by abrasion from car tires or clothing [23, 24] and can release chemical additives [25] in the body [26]. It seems likely that these effects are also relevant to humans [27].

While the major constraints of NMP in- and uptake occur in the lung and intestine, the primary barrier of humans to the environment, in fact, is the skin. Nano- and microplastic are part of many pharmaceutical and cosmetic formulations, and ambient air polymeric particles frequently settle on the skin with so far unknown consequences. Therefore, dermal NMP uptake is another significant route, motivating the examination of NMP effects on skin cells in our study. To this end, we investigated the uptake and biological consequences of fluorescently-labeled polystyrene NMP in primary cells freshly isolated from murine skin. We hypothesized to identify structural changes in epidermal and dermal skin cells, which could be related to the dysregulation of molecular processes induced by intracellular stress responses and intracellular plastic particle accumulation. This was tested using polystyrene plastic particles of five different sizes and exposing the cells either once (acute dose = short-term) or repetitively (chronic dose = long-term) to the NMP. Oxidative stress and major effects on a range of critical biological processes and pathways were observed using fluorescent microscopy, flow cytometry, and gene and protein expression analysis.

## Materials and methods

### Preparation of cell culture

The study was conducted in accordance with the regulations of the local ethics committee of the State of Mecklenburg-Vorpommern (Rostock, Germany; Az.: 7221.3–1-044/16) and the guidelines for care and use of laboratory animals. It was performed according to the recommendations of Good Laboratory Practice and within the guidelines and regulations of animal care and experimentations such as the ARRIVE guidelines in SKH1-hr hairless immunocompetent mice (Charles River Laboratories, Germany). Primary cells were isolated from healthy mouse skin by enzyme-mediated removal and digestion of the epidermal and dermal layers according to the recommendations of an epidermis dissociation kit (Miltenyi Biotec, Germany). To this end, the cell suspension was homogenized in gentleMACS C tubes using a gentleMACS dissociator for obtaining live cells (Miltenyi Biotec, Germany). Then, it was passed through 70 µm MACS SmartStrainers to separate individual cells. The resulting mix of skin cells, including dermal fibroblasts and epidermal keratinocytes, were cultured over 10 days in Eagle's Minimum Essential Medium (EMEM; PromoCell, Germany) supplemented with 10% fetal bovine serum, 1% penicillin/streptomycin, and L-glutamine (Sigma-Aldrich, Germany) in a humidified incubator at 37 °C with 5% CO_2_. In our experimental setups, early passages from 1 to 5 were used [28].

### Polymer particle design and exposure to biological material

The NMP used in this study were the fluoresbrite (FB) polystyrene microspheres 0.2 (YG, 0.2 µm, catalog number 07304), 1.0 (YG, 1 µm, catalog number 17154), 2.0 (red, 2 µm, catalog number 19508), and 6.0 (red, 6 µm, catalog number 19111) (all Polyscience, USA), and polymer microspheres mix (red, 1–5 µm, catalog number FMR-1.3; Cospheric, USA) in aqueous suspension. The fluorescence dyes were incorporated in the core of the NMP to avoid dye-cell interactions. Stocks were sonicated in a sonicator bath and thoroughly vortexed before use. Environmentally relevant concentrations of NMP are between 1.6 µg/mL, found in human blood samples [29], and 100 to 1000 µg/mL to facilitate studies on biological effects [30]. In line with the literature and own experimental findings [31], 10 × concentrated NMP suspensions were added, reaching 1 × final concentration of 100 µg/mL in PBS. Skin cell monolayers (70% confluence) were incubated with NMP in corresponding cell culture dishes ranging from 6 cm^2^-dishes for generating RNA and protein lysates (5 × 10^5^ cells) over 12-well plates (1.5 × 10^5^ cells) for immunofluorescence-imaging to 96-well plates (5 × 10^3^ cells) for metabolic activity and viability measurements. Cytoplasmic and intracellular membrane structures were stained with a blue fluorescent dye (CellBriteBlue; Biotium, San Fransisco, USA) and visualized along with fluorescent NMP uptake into the skin cells using a confocal laser scanning high-content imaging system and several z-stack image series integrated into maximum intensity projections for improved visibility (Operetta CLS; PerkinElmer, Germany). Quantification, however, was based on true 3D object quantification, a recent feature added to Harmony 4.9 software (PerkinElmer, Germany) package, as we had described recently [31].

### Analysis of intracellular ROS and thiol content

The DCF-DA method was applied to measure intracellular oxidative stress level. 5 × 10^3^ skin cells were pretreated with various NMP and incubated for 24 h in a 96-well plate. Next, cells were washed with PBS and treated with H_2_DCF-DA (final concentration 25 µM; ThermoFisher, Germany) at 37 °C for 1 h. H_2_O_2_ (100 µM) was used as a positive control. The fluorescence intensity correlates with specific enzymatic activity in the presence of ROS and was assessed in a microplate reader (F200; Tecan, Switzerland) at λ_ex_ 485 nm and λ_em_ 525 nm. The thiol content was analyzed using flow cytometry after adding a thiol-detecting reagent (5 µM; ThiolTracker violet; Thermo Fisher Scientific, Bremen, Germany)) to NMP-treated skin cells. Additionally, the granularity of MP-treated skin cells was determined by flow cytometry in the side scatter (SSC) and compared to untreated cells (for details, see below).

### Analysis of cellular metabolism, viability, and apoptosis

For the NADPH-based resazurin (7-hydroxy-3H-phenoxazin-3-one 10-oxide) assay, 5 × 10^3^ skin cells were exposed to NMP. Resazurin (100 µM; Alfa Aesar, Germany) is reduced to fluorescent resorufin by metabolically active cells, and the fluorescence was determined after 24 h using a microplate reader at λ_ex_ 535 nm and λ_em_ 590 nm. The viability of skin cells was analyzed via live-dead discrimination [32]. Briefly, cells were stained seven days after first NMP exposure with 2 µM Calcein-AM, 1 µM of propidium iodide (PI), and 5 µM of Hoechst 33342 (Life Technologies, USA), and imaged using fluorescence microscopy (Axio Observer Z.1; Zeiss, Germany). Additionally, cells were collected in FACS tubes and washed three times with cold FACS washing buffer (Miltenyi Biotec, Germany). Cells were stained with caspase 3/7 detection reagent (ThermoFisher, Germany) and 4′,6-diamidine-2-phenylindole (DAPI, final concentration 1 µM; BioLegend, The Netherlands) at 37 °C for 30 min. After washing with cold FACS washing buffer, samples were measured using flow cytometry (CytoFLEX S/LX; Beckman-Coulter, Germany) and analyzed using Kaluza software 2.1 (Beckman-Coulter, USA).

### Multiplex cytokine analyses

According to the manufacturer's instructions, the inflammatory secretion profile was measured in supernatants of SKH1-derived skin cells cultured with NMP (up to four weeks) using multiplex cytokine detection technology (LegendPlex; BioLegend, The Netherlands). The bead-based sandwich immunoassay was measured using flow cytometry (CytoFLEX S; Beckman-Coulter, Germany) targeting tumor necrosis factor-alpha (TNFα), interferon (IFN) α and γ, monocyte chemotactic protein (MCP) 1 (or CCL2), and nine interleukins (IL1β, IL6, IL10, IL12p70, IL17A, IL18, IL23, and IL33). Appropriate data analysis software (BioLegend, USA) was utilized for target quantification.

### RNA extraction and real-time PCR to quantify mRNA gene expression

After lysis of NMP-treated cells in RNA lysis buffer, total RNA was isolated according to the manufacturer's instructions (Bio&Sell, Germany), and mRNA expression levels were determined by quantitative PCR (qPCR). Briefly, 1 μg of RNA was transcribed into cDNA, and qPCR was conducted in duplicates using SYBR green mix (Roche Diagnostics, Switzerland). Gene-specific primers (Additional file 1: Table S1) were used (BioTez, Germany). The housekeeping genes *GAPDH* and *RPL13A,* whose expression was unaffected by NMP exposure, were used as an internal normalization control. Gene expression was analyzed using the ^ΔΔ^CT method.

### Protein extraction and WES analyses to quantify protein expression

For protein extraction, cells were lysed in RIPA buffer containing protease and phosphatase inhibitors (cOmplete Mini, phosSTOP, PMSF; Sigma-Aldrich, Germany). Protein expression levels of the Nrf2, HO-1, Nqo1, Cat, Sod1, β-catenin, E-cadherin, Fak, Vcl, and β-actin were determined using corresponding antibodies (Cell Signaling, Germany), and the *WES* system and *Compass* software (both ProteinSimple, Germany) according to the manufacturer's instructions. GAPDH or β-actin served as housekeeping control, and band intensities were displayed as fold change to the corresponding control.

### Immunofluorescence imaging to study the bioaccumulation and translocation of NMP

Skin cells were seeded on glass coverslips and exposed to NMP 24 h later. Uptake and internalization of polystyrene NMP were further studied with a live-cell high-content imaging system (Operetta CLS; PerkinElmer, Germany), and algorithm-driven image quantification was performed using dedicated imaging software (Harmony 4.9; PerkinElmer, Germany). For immunofluorescence microscopy of protein targets, samples were fixed in 4% paraformaldehyde (Sigma-Aldrich, Germany) for 20 min, washed, and permeabilized with Triton X-100 (0.01% in PBS; Sigma-Aldrich, Germany). Next, samples were incubated with primary antibodies targeting Bcl2, γH2AX, Nrf2, β-catenin, collagen I, Vcl (all Cell Signaling, Germany), and FITC- (fluorescein isothiocyanate) or FR- (flash red) labeled phalloidin for actin cytoskeleton staining, followed by staining with secondary antibodies conjugated to Alexa Fluor 488 for red NMP or Alex Fluor 594 for green NMP, respectively (Life Technologies, Germany). The terminal deoxynucleotidyl transferase dUTP nick end labeling (TUNEL) staining was performed using the *DeadEnd Fluorometric TUNEL* System according to the manufacturer's instructions (Promega, Germany). DAPI was used to counterstain nuclei*.* Samples were mounted onto glass microscope slides using mounting medium (VectaShield; Biozol, Germany) before fluorescence microscopy using an Axio Observer Z.1 (Zeiss, Germany).

### Statistical analysis

Data are presented as mean + S.E.M. of at least three independent experiments. Graphing, statistical analysis, and principal component analysis (PCA) were performed using prism 9.3 (GraphPad Software, USA). The number of experiments and type of statistical analysis are given in the figure legends. The online software biorender.com was used to create some of the schemes. *Student*'s t-test was used for comparison between two groups, and one-way analysis of variances (ANOVA) was used for multiple group comparison, with *p*-values indicated by *p ≤ 0.05, **p ≤ 0.01, and ***p ≤ 0.001. Gene and protein expression were Pearson correlated against the NMP size using TipCo Spotfire 7.8 software (PerkinElmer, Germany).

## Results

### Uptake of NMP in an acute and chronic exposure primary skin cell culture model

**Fig. 1:**
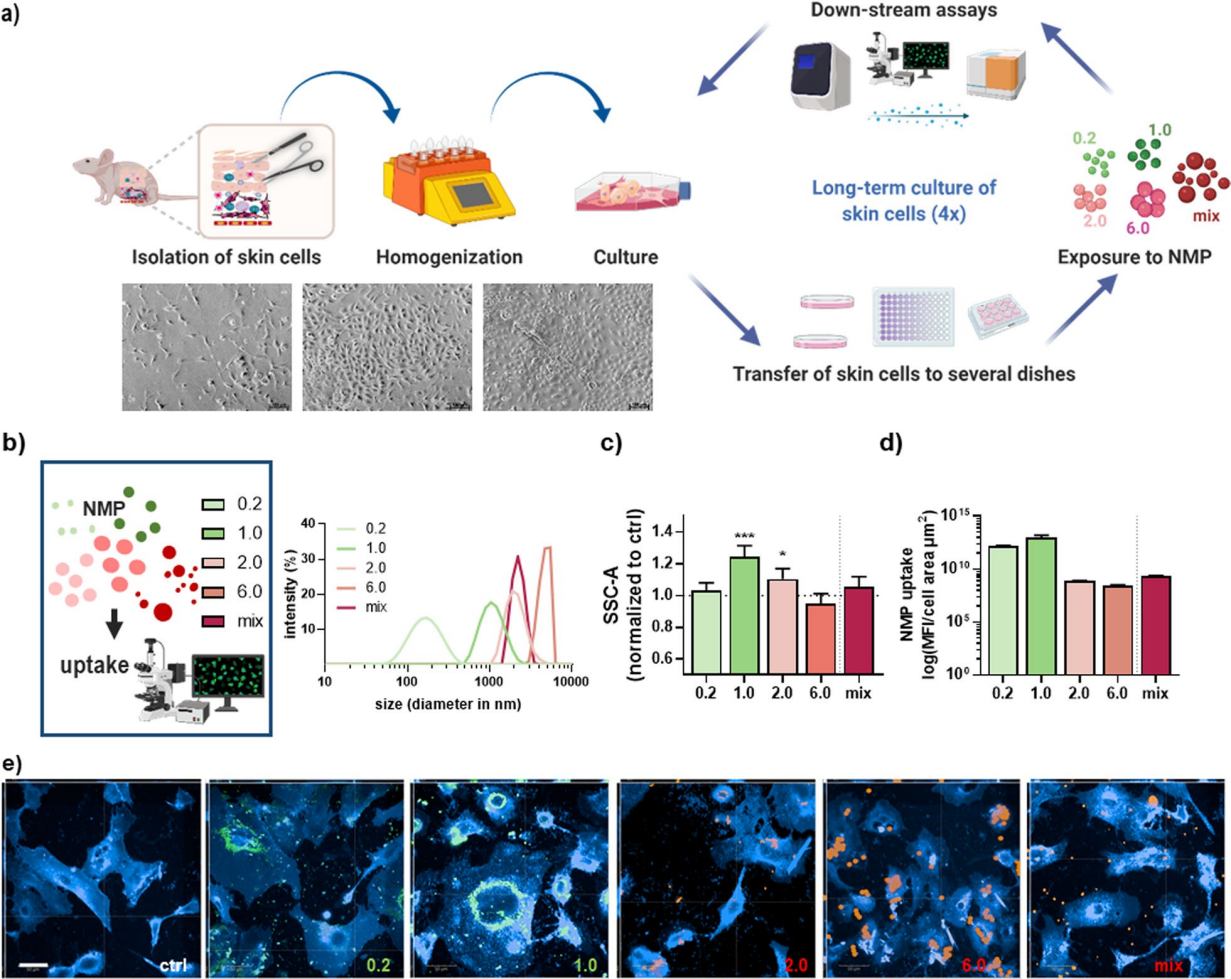
Study design and uptake of fluorescently labeled polystyrene NMP in skin cells. **a** After homogenization of murine skin tissue, skin cells (i.e., mix of primary keratinocytes and dermal fibroblasts) were cultivated and incubated with NMP over several weeks; at the end of every week (d7, d14, d21, d28), downstream analyses were performed including determination of NMP uptake, secretion profiling, gene and protein expression of selected targets. Representative images show skin cells in the brightfield channel. **b** Study scheme and dynamic light scattering size verification of several polystyrene NMP with fluorescent labeling ranging from 0.2 µm to 6 µm. **c** Flow cytometry analysis of cells incubated with NMP and quantification of side-scatter signals from individual cells (SSC). **d** Algorithm-based image analysis and calculation of NMP uptake. **e** 50 z-stack maximum intensity projection of murine skin cells with fluorescent membrane label (blue) and fluorescent particles inside as well as outside of the cell. All data were normalized to control cells (i.e., 1.0) non-exposed to NMP. Statistical analysis was done by unpaired, two-tailed Student's *t* test (n > 3) with *p ≤ 0.05 and ***p ≤ 0.001. Scale bars are 100 µm (**a**) and 50 µm (**e**)

The use of skin cells in this study as relevant cells for the skin barrier can provide important information about the toxicity-related parameters of NMP. Therefore, freshly isolated cells from murine skin, such as dermal fibroblasts and epidermal keratinocytes, were cultivated over several weeks. NMP were added once (acute) or regularly every week to the skin cells, with the endpoint reached after one week (acute) and four (chronic) weeks, respectively. Several biological responses were analyzed, such as uptake and bioaccumulation, toxicity, secretion profiles, and oxidative stress (Fig. 1a). Advantages of polymeric NMP microspheres are the precise control of particle characteristics, such as the possibility of an early surface modification, flexibility in manufactured size, and the use of fluorescence labeling. In the present study, green or red fluorescence-labeled spheric polystyrene NMP were used. The different sizes (0.2 µm, 1.0 µm, 2.0 µm, 6.0 µm, and mixed NMP at around 1–5 µm) were validated by measurements with a light scattering system (Fig. 1b). Particle uptake was associated with increased intracellular vesicles, leading to higher light reflection. Hence, we quantified light scattering in NMP-treated cells using flow cytometry. Particularly the 1.0 µm and 2.0 µm particle exposure conditions significantly increased side scatter signals (Fig. 1c). The cellular NMP uptake was further quantified, indicating a higher relative accumulation of smaller over larger particles compared to particles with the same fluorescence dye (Fig. 1d). After staining of the cytoplasmic membrane and intracellular membrane structures (blue), the cellular distribution in dependence on the size of NMP was imaged by fluorescence microscopy, pointing out that polystyrene NMP accumulated in the cytoplasm around the cell nucleus (Fig. 1e, Additional file 1: Fig. S1).

### NMP exposure affected ROS formation, viability, and apoptosis in primary skin cells

**Fig. 2:**
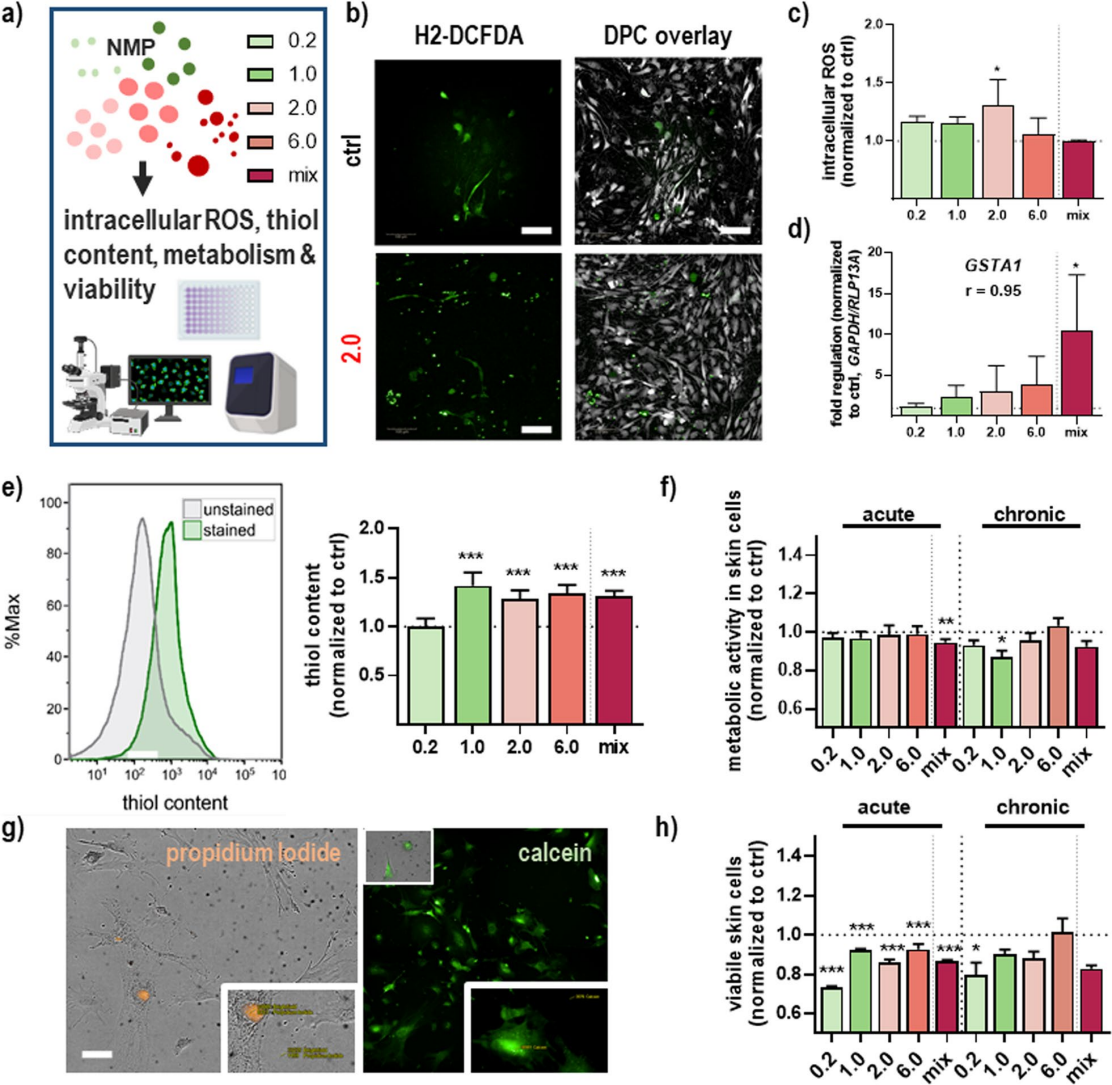
Intracellular ROS, thiol content, metabolic activity, and viability in NMP-treated skin cells. **a** Study scheme. **b** Representative images of H_2_-DCF-DA staining in untreated (upper panel) and NMP (2.0)-treated skin cells (lower panel). **c** Intracellular ROS levels were determined by flow cytometry. **d** qPCR of glutathione-S-transferase A1 (*GSTA1*) expression after NMP uptake. **e** Representative histogram of intracellular thiol content fluorescence intensity of stained and unstained cells using flow cytometry, and quantification of thiol content. **f** Cellular metabolic activity after acute and chronic NMP exposure. **g** Representative images of skin cell viability showing nuclear propidium iodide (orange) and cytosolic calcein staining (green). **h** Quantification of viable cells after acute and chronic NMP exposure. Scale bars are 100 µm (**b**) and 50 µm (**g**). All data were normalized to control cells (i.e., 1.0) non-exposed to NMP. Statistical analysis was done by unpaired, two-tailed Student’s *t* test (n > 3) with *p ≤ 0.05, **p ≤ 0.01, and ***p ≤ 0.001

The uptake of small NMP has been linked to elevated intracellular reactive oxygen species (ROS) levels that can cause acute or long-term toxic effects. Hence, we investigated ROS formation (Fig. 2a) using the general ROS indicator H2DCF-DA (Fig. 2b), indicating a significant increase for 2.0 µm NMP (Fig. 2c). The alpha class of glutathione S-transferase (GST) enzymes exhibit glutathione peroxidase activity, thereby protecting the cells from ROS and peroxidation products. To this end, the *GSTA1* expression was determined using qPCR, showing an increased expression level and catalytic process of GST in the phase II detoxification process in response to NMP treatment. Moreover, we found a strong correlation and direct relation between particle size and expression level (r = 0.94) for GSTA1 (Fig. 2d). Generally, for identifying statistical relations and a possible linear association between NMP sizes and observed biological effects, the Pearson correlation coefficients for all gene (upper tables) and protein expression (lower tables) levels were determined for acute and chronic NMP exposure regimens, respectively (Table 1). Next, we measured the thiol content in NMP-treated skin cells, showing a significant increase in all NMP sizes investigated, except for 0.2 µm (Fig. 2e). When assaying the metabolic activity of the cells, no substantial decline was found for either one dose or repetitive exposure (Fig. 2f). In live-dead analysis (Fig. 2g), the low amplitude of long-term toxic effects was similar, except for a single NMP exposure (Fig. 2h).

**Table 1:**
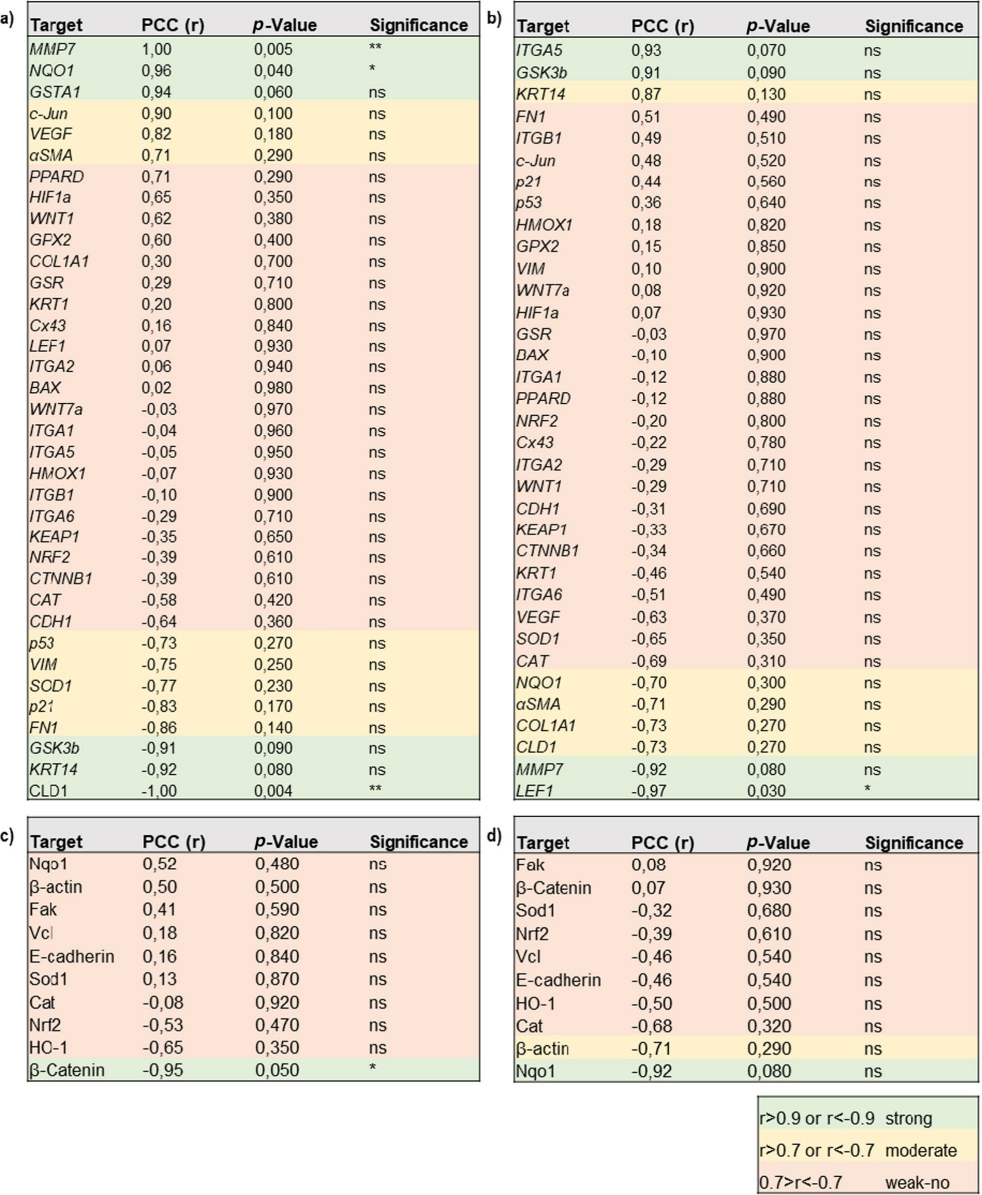
Pearson correlation coefficients for all gene expression (upper tables) and protein expression (lower tables) analyses performed in this study for acute (a) and chronic (b) plastic exposure regimens for identifying statistical relations between plastic particle sizes and biological effects observed, sorted for descending r values

**Fig. 3:**
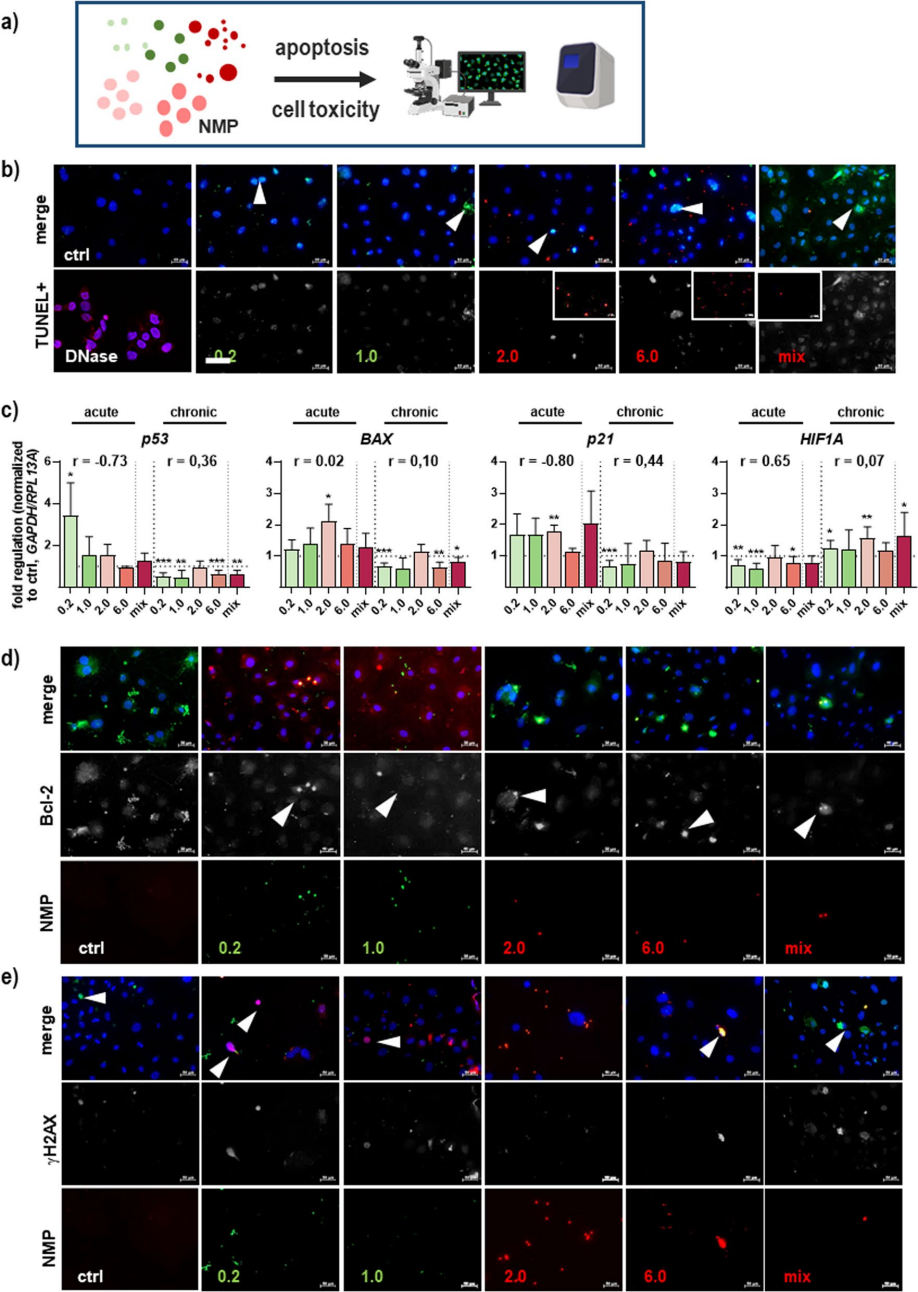
Apoptosis in NMP-treated skin cells. **a** Study scheme. **b** Skin cells were grown on glass coverslips, NMP-treated, fixed, and subjected to fluorescent staining to determine the expression and distribution of TUNEL-positive apoptotic cells. As positive control, skin cells were incubated with DNase (violet). **c** Quantification of apoptosis-related *p53* and *BAX* mRNA, cell-cycle-related *p21,* and *HIF1A* mRNA using qPCR. Data were normalized to *GAPDH/RLP13A* and untreated controls (ctrl) and presented as mean + SEM. Statistical analysis was done by unpaired, two-tailed Student’s *t* test (n > 3) with *p ≤ 0.05, **p ≤ 0.01, and ***p ≤ 0.001. **d** Distribution and expression of Bcl-2 protein and **e** H2AX phosphorylation (γH2AX) were analyzed using immunofluorescence imaging of NMP-treated skin cells. Nuclei were stained with DAPI (blue). Scale bars are 50 µm

To investigate the nature of cell death (Fig. 3a), the TUNEL assay was used to detect DNA fragmentation generated during apoptosis, showing a modest increase of TUNEL-positive cells following NMP exposure independent of their size (Fig. 3b). Moreover, NMP treatment significantly altered apoptosis- and cell-cycle-related gene transcriptional expression profiles. The expression pattern of the tumor suppressor protein *p53*, Bcl-2-associated X protein (*BAX*), a cofactor of p53, and cell cycle-related gene p21 tended to increase after acute NMP exposure. However, these were rather down-regulated with repeated NMP exposure compared to controls. In contrast, the observed decrease of the hypoxia-inducible factor 1α (*HIF1A*) expression after single incubation has reversed with increasing duration of NMP exposure, indicating a regulation through a redox-sensitive mechanism (Fig. 3c). The Bcl-2 protein family plays essential roles in the regulation of intrinsic, mitochondrial apoptotic cell pathway by either inhibiting (anti-apoptotic) or inducing (pro-apoptotic) apoptosis [33]. Thus, we investigated Bcl-2 protein expression in untreated skin cells showing stronger expression compared to NMP-treated cells (Fig. 3d). Increased Bcl-2 expression has been associated with increased resistance of skin cells to DNA damage-induced cell death [34]. As highlighted by immunofluorescence staining, we observed a more than twofold accumulation of phosphorylated histone 2A complex (γH2AX) in the cell nucleus after NMP exposure (Fig. 3e).

### NMP exposure altered the secretion profile in primary skin cells

**Fig. 4:**
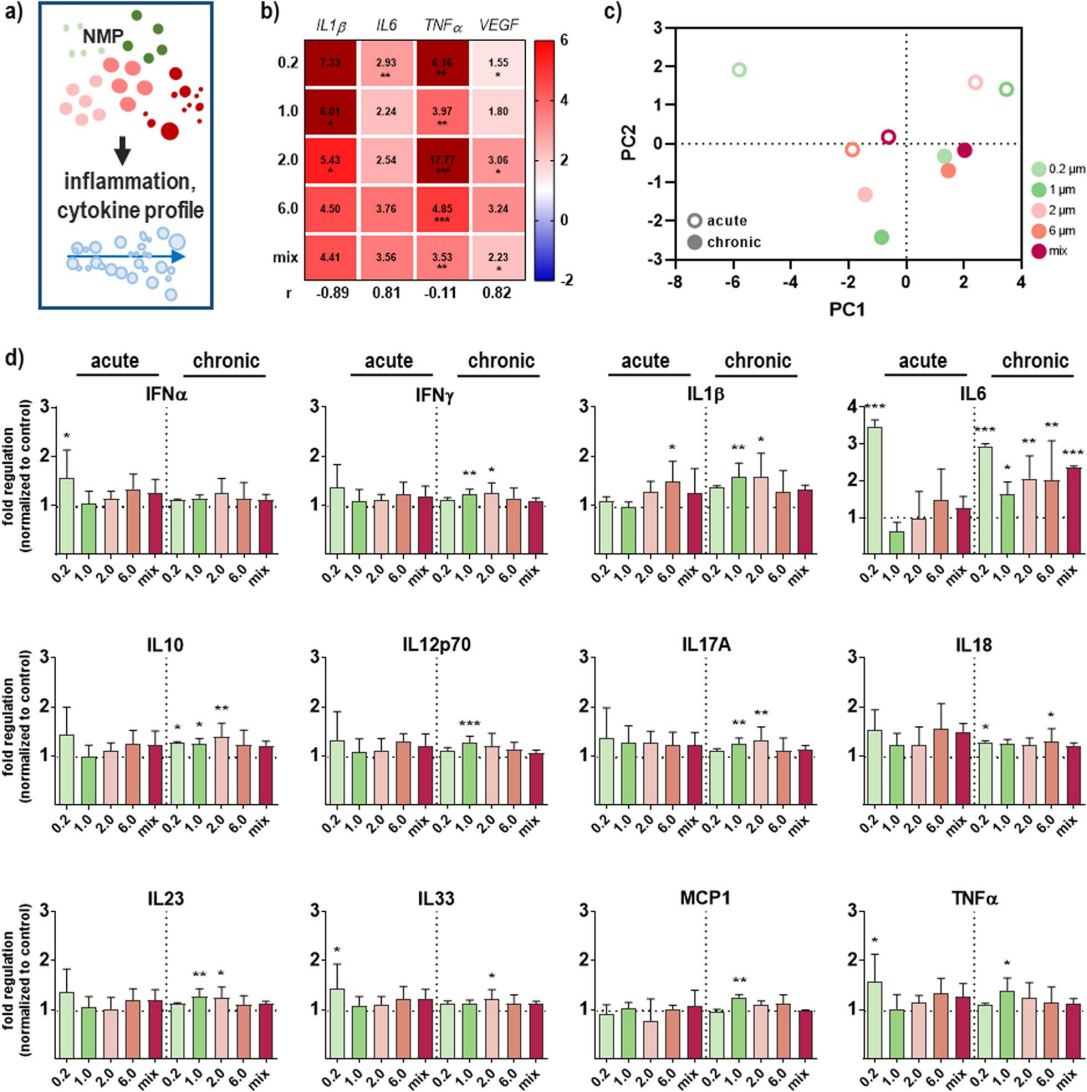
Secretory response of NMP-treated skin cells. **a** Study scheme. **b** After NMP exposure, expression values of IL1β, IL6 TNFα, and VEGF were determined by qPCR and shown in a heat map. **c** Principal component analyses (PCA) of normalized cytokine and chemokine levels across all NMP sizes revealed PC2 to separate between acute and chronic stress response. **c** Multiplex chemokine and cytokine release quantification in skin cell supernatants after acute and chronic NMP exposure. Data were normalized to *GAPDH/RLP13A* and untreated controls (ctrl) and presented as mean + SEM. Statistical analysis was done by one-way ANOVA (n ≥ 3 for (**b**; n ≥ 9 for **d**) with Dunnett's multiple comparisons test (*p ≤ 0.05, **p ≤ 0.01, and ***p ≤ 0.001)

Since it is important to know whether pro- or anti-inflammatory reactions are more likely to be observed when exposed to NMP, our goal was to identify the secretory response of NMP-treated skin cells (Fig. 4a). First, we focused on examining the induction of pro-inflammatory cytokine expression through quantitative real-time PCR (qPCR). NMP exposure caused a significant upregulation of TNFα expression, indicating an NMP size-independent response. Interleukin 1β (IL1β), another mediator of an inflammatory response, has shown the same expression profile as TNFα. A slight upregulation was observed for interleukin 6 (IL6) after NMP treatment in all experimental groups (Fig. 4b). More important, secreted chemokines and cytokines were measured directly in cell culture supernatants. Principal component analyses (PCA) of normalized cytokine and chemokine levels across all NMP sizes revealed PC2 to separate acute and chronic stress responses (Fig. 4c). In particular, IL6 was significantly increased in NMP-treated skin cells. At the same time, the strong regulation found for TNFα and IL1β in qPCR results was not confirmed. In general, chronic NMP exposure had a more pronounced effect on the secretion profile than single exposure (Fig. 4d).

### NMP exposure affected redox homeostasis and Nrf2 stress signaling

**Fig. 5:**
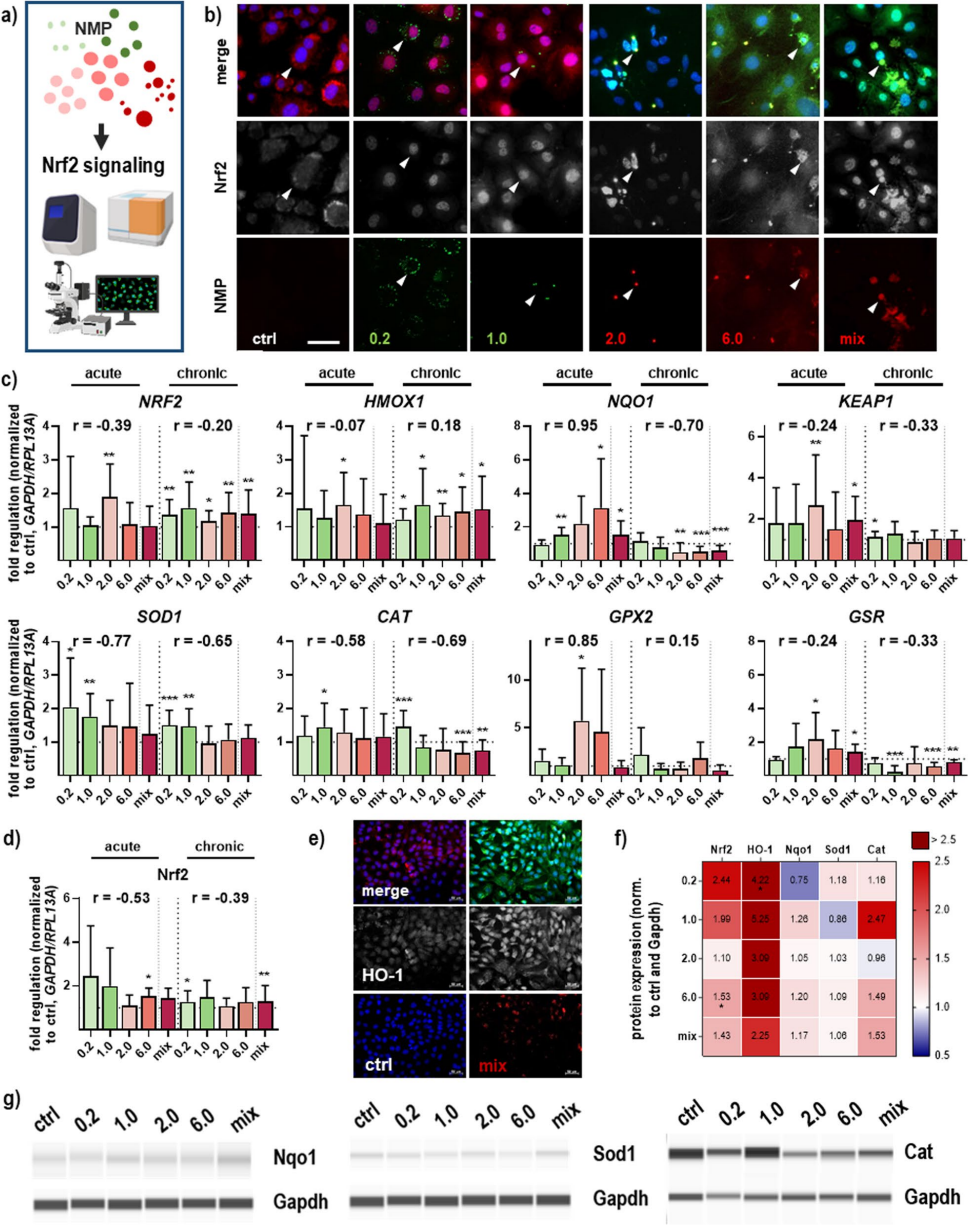
Activation of Nrf2 signaling in skin cells following NMP uptake. **a** Study scheme. **b** Skin cells were grown on glass coverslips, incubated with NMP, fixed, and subjected to fluorescent staining to determine the expression and distribution of the Nrf2 protein (red or green, arrowheads showed nuclear staining in contrast to ctrl). **c** Quantitative mRNA expression analyses of the Nrf2 signaling pathway and its downstream targets. **d** Nrf2 protein levels. **e** Distribution and expression of HO-1 after nuclear Nrf2 translocation and activation were observed using immunofluorescence labeling in skin cells after mix-NMP uptake. The cell nuclei were stained with DAPI (blue). Scale bar is 50 µm. **f**–**g** Protein expression heatmap of Nrf2 and its downstream targets determined by WES (**f**), and representative WES images of Nqo1, Sod1, and catalase (**g**). For qPCR and WES, data were normalized either to *GAPDH/RLP13A* or Gapdh, respectively, and untreated controls (ctrl), and presented as mean + SEM. Statistical analysis was done by unpaired, two-tailed Student’s *t* test (n > 3) with *p ≤ 0.05, **p ≤ 0.01, and ***p ≤ 0.001

The protein distribution and quantification of the gene transcript and protein expression following NMP exposure were explored by immunofluorescence microscopy, qPCR, and WES, indicating that the nuclear factor-E2-related transcription factor 2 (Nrf2) pathway was affected in our primary skin cell model (Fig. 5a). We observed a strong nuclear translocation of Nrf2 independently of NMP size, indicating an activation of Nrf2 signaling following NMP exposure (arrowheads in Fig. 5b). Antioxidant responsive element (ARE)-driven genes include direct stress-responsive genes (*e.g.*, heme oxygenase (HMOX) 1; NAD(P)H dehydrogenase [quinone] (NQO) 1), antioxidant (e.g., glutathione peroxidase (GPX) 2; superoxide dismutase (SOD) 1; catalase, CAT), and detoxifying enzymes (glutathione reductase, GSR), showing significantly expression modulation to NMP exposure in a size-dependent fashion. Several downstream targets of Nrf2 (Fig. 5c) are mainly upregulated after acute NMP exposure. After repeated NMP exposure, mRNA levels of *NRF2, HMOX1, KEAP1*, and *SOD1* were increased or at the same level as in untreated cells. By contrast, *NQO1*, *CAT, GPX2,* and *GSR* expression levels decreased compared to the controls in a time-dependent manner (Fig. 5c). In this regard, the protein expression level of Nrf2 was only slightly increased upon NMP exposure (Fig. 5d). Additionally, protein distribution and antibody-labeling of heme oxygenase (HO-1) validated increased (cytosolic) expression of HO-1 by fluorescence microscopy (Fig. 5e and Additional file 1: Fig. S2). In contrast, all other Nrf2 down-stream proteins such as Nqo1, Sod1, and Cat were only slightly increased in NMP-treated skin cells after acute NMP exposure (Fig. 5f). Additionally, representative images of protein expression were shown for Nqo1, Sod1, and Cat using WES analysis (Fig. 5g).

### NMP exposure modulated β-catenin and its target gene expression in murine skin cells

**Fig. 6:**
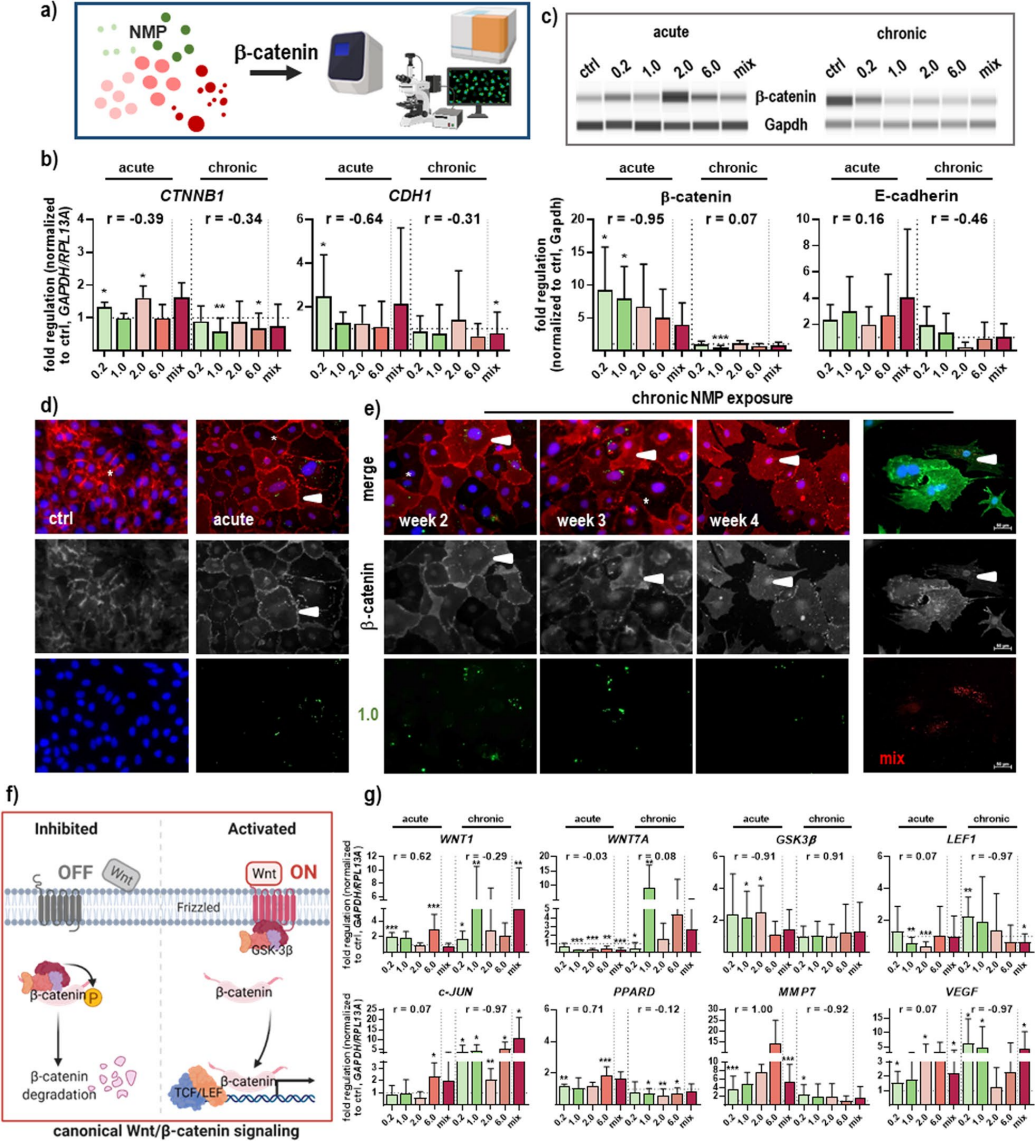
Effects on β-catenin signaling and target genes following NMP uptake in skin cells. **a** Study scheme. **b** qPCR-based gene expression analysis of *CTNNB1* and *CDH1*. **c** Representative WES-images of β-catenin protein expression after acute (left) and chronic (right) NMP exposure (upper panel), and WES-based protein expression analysis of β-catenin and E-cadherin. **d**–**e** Expression and distribution of β-catenin after acute exposure to 1.0 µm NMP compared to ctrl (left panel) or chronic exposure after 2, 3, and 4 weeks (right panel) to 1.0 and NMP mix, respectively. The cell nuclei were counterstained with DAPI (blue), the cytoplasmic distribution of β-catenin was marked with a star, and the nuclear β-catenin was marked with arrowheads. Scale bars are 50 µm. **f** Scheme of initiation of downstream signaling by binding of Wnt proteins to a frizzled family receptor. The signal is propagated via β-catenin and ends with a change in transcription of target genes (*e.g.*, activation of Wnt signaling; right). Inhibition of Wnt signaling activation after phosphorylation and degradation of β-catenin (left). **g** qPCR-based gene expression analysis of *WNT1/7a*. Differential expression of β-catenin target genes (e.g., *LEF1, c-JUN, PPARD, GSK3β*, *MNMP7*, *VEGF*). For qPCR and WES, data were normalized either to *GAPDH/RLP13A* or Gapdh, respectively, and untreated controls (ctrl), and presented as mean + SEM. Statistical analysis was done by unpaired, two-tailed *Student*'s *t* test (n ≥ 3) with *p < 0.05, **p ≤ 0.01, and ***p ≤ 0.001

Β-catenin and E-cadherin are molecules that bind intracellularly to the adhesion molecules and create a stabilizing connection to the actin cytoskeleton (Fig. 6a). qPCR and WES analysis showed a substantial increase in β-catenin (*CTNNB1*) gene and protein level, and a slight increase in E-cadherin (*CDH1*) expression after acute NMP exposure, in contrast to chronic treatment (Fig. 6b–c). Interestingly, we observed this incubation time-dependent increase in accumulation and nuclear staining using immunofluorescence, demonstrating an activation of the β-catenin pathway. The subcellular location of β-catenin showed membraneous, cytoplasmic, and nuclear expression following exposure to 1.0 µm NMP-treated cells over four weeks (arrowheads, Fig. 6d–e). Stronger membraneous staining at cell–cell contacts was mainly observed in skin cells where NMP were not incorporated (star, Fig. 6d). Exposure with all other NMP sizes investigated showed similar results in acute- (Additional file 1: Fig. S3) and chronic-treated skin cells (Fig. 6e). In the presence of Wnt ligands (*e.g.*, Wnt1, Wnt7a), *CTNNB1* is not ubiquitinated but accumulates in the nucleus, where it acts as a coactivator for transcription factors of the T-cell/lymphoid enhancer-biding factor (TCF/LEF) family. In the absence of Wnt ligands, glycogen synthase kinase (GSK) 3β promotes the phosphorylation of β-catenin at key Ser/Thr residues [35], targeting it for degradation through the ubiquitin-ligase pathway (Fig. 6f). Hence, we further examined Wnt genes expression in skin cells using qPCR, showing a correlation between the localization status of β-catenin and expression levels of Wnt1 and Wnt7a. Both molecules were strongly upregulated after chronic NMP exposure, independent of particle size. Additionally, qPCR was performed to efficiently compare the expression profiles of the modulators of this pathway between NMP-treated cells and matched controls. The analyses revealed that the mRNA level of GSK3β and LEF1 were consistent with previous data showing a significant decrease of *GSK3β* but an increase of *LEF1* mRNA in single-NMP-treated skin cells. After prolonged NMP exposure, *GSK3β* was comparable between NMP-treated cells and controls, whereas *LEF1* mRNA was increased in the NMP-treated skin cells (upper panel, Fig. 6g). To further determine the role of c-JUN in canonical Wnt signaling as an activator downstream of β-catenin stabilization, we investigated its expression level showing a solid increase of *c-JUN* after prolonged exposure in NMP-treated skin cells. The lipid-sensitive nuclear peroxisome proliferator-activated receptor (PPAR) δ is widely distributed in the nucleus of epithelial lineages from keratinocytes in the skin [36]. PPARD mRNA expression increased slightly after acute treatment but significantly decreased after prolonged NMP exposure. The matrix metalloproteinase (MMP) 7 is upregulated in response to β-catenin-mediated LEF1-dependent transcription in NMP-treated skin cells. Other molecules, such as the level of vascular endothelial factor (*VEGF*) mRNA, were significantly elevated in NMP-treated skin cells (lower panel, Fig. 6g).

### NMP exposure affected actin cytoskeleton, barrier-modulating structures, and intermediary filaments

**Fig. 7:**
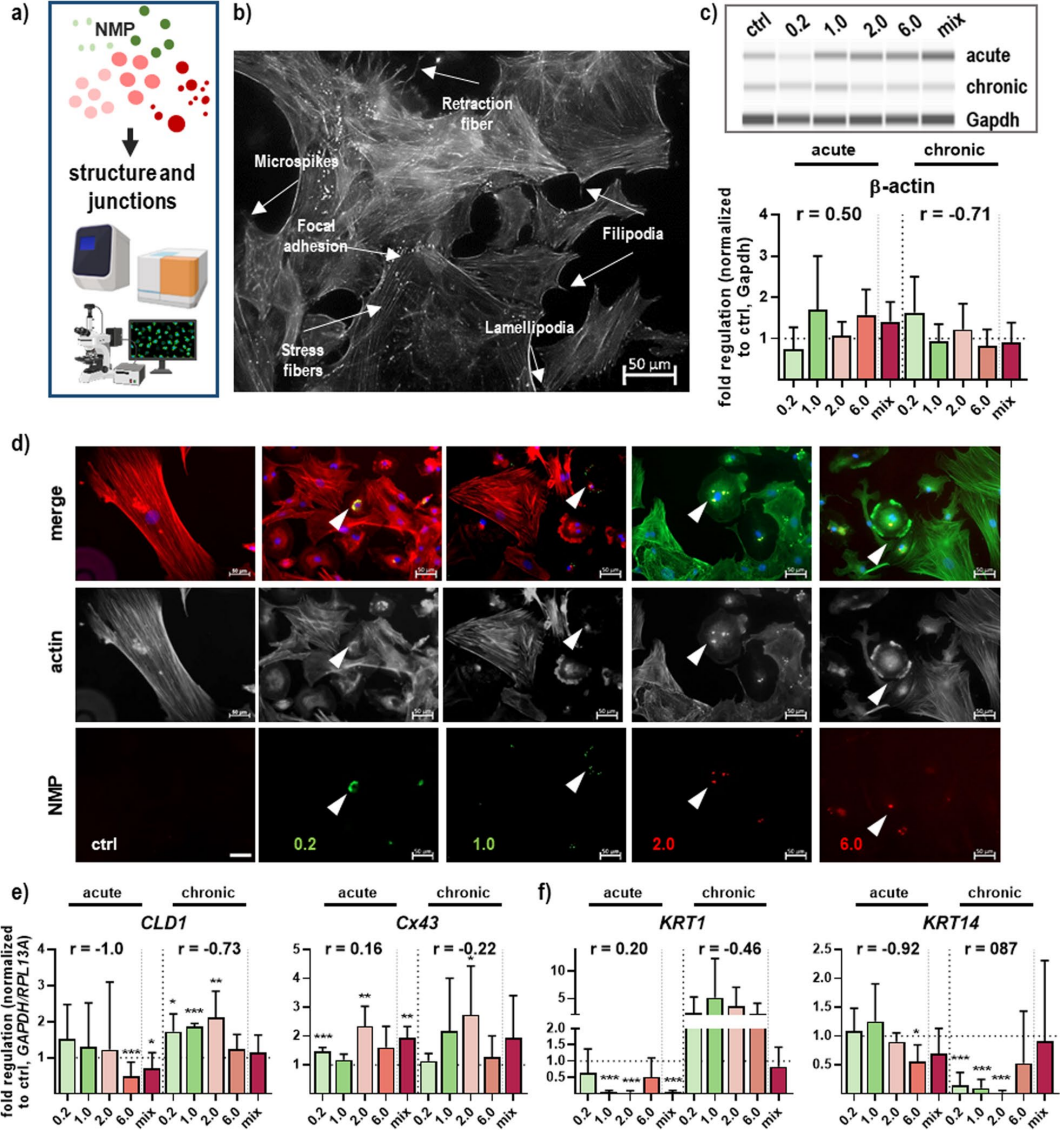
Structural and junctional proteins after skin cell NMP treatment. **a** Study scheme. **b** Components of the actin cytoskeleton in representative fibroblast-like skin cells. **c** Representative WES images and quantification of β-actin protein expression. **d** Skin cells were grown on glass coverslips, incubated with selected NMP, fixed, and subjected to fluorescent labeling of actin stress fibers using FITC- (green) or FlashRed-phalloidin (red, arrowheads showed transient breakdown of cytoskeleton after NMP uptake), respectively, with or without nuclear counterstaining (DAPI, blue). Scale bars are 50 µm. **e**–**f** mRNA expression levels of junctional proteins (*CLD1, Cx43*; **e**) and epidermal proteins (*KRT1/14*; **f**) were quantified after single and repeated NMP exposure by qPCR in skin cells. For qPCR and WES, data were normalized either to *GAPDH/RLP13A* or Gapdh, respectively, and untreated controls (ctrl). Results were presented as mean + SEM. Statistical analysis was done by unpaired, two-tailed Student’s *t* test (n > 3) with *p ≤ 0.05, **p ≤ 0.01, and ***p ≤ 0.001

One role of the actin cytoskeleton is regulating the β-catenin / E-cadherin complex between adherent cells. Thus, we investigated structural and junctional target expression and distribution following NMP exposure (Fig. 7a). In unaffected skin cells, components of the actin cytoskeleton were identified via immunofluorescence staining, as shown in representative fibroblast-like cells. Individual components of the actin cytoskeleton are marked as well-defined bundles of stress and retraction fibers, lamellipodia, filipodia, focal adhesion, and microspikes (Fig. 7b) reinforcing adhesion sites between cells or between a cell and the extracellular matrix (ECM), maintaining and changing the cell shape, and defining the mechanical properties of the cell surface [37]. While the β-actin expression level of *ACTB* mRNA was not changed upon NMP exposure (Fig. 7c), we found a strong NMP effect on the cytoskeletal actin filaments together with changes in cell phenotype. The transient breakdown of the actin cytoskeleton was associated with an absence of long actin stress fibers, lamellipodia, and filopodia, and a more diffuse intracellular staining in those NMP-treated skin cells that had accumulated NMP (arrowheads, Fig. 7d). Integral proteins of epidermal tight junctions were mainly upregulated after repeated NMP treatment, as shown for claudin 1 (*CLDN1*) mRNA. Additionally, gap junctions, as channels for intercellular cell–cell communication (GJIC), are mainly formed by connexin (Cx) 43. We found a marked and modest upregulation for *Cx43* in dependence of single or repeated NMP treatment, respectively, indicating a general effect of NMP on GJIC (Fig. 7e). The gene expression of keratin 1 (*KRT1*), which is found in keratinized structures of the epidermis, was dramatically enhanced after prolonged treatment. In contrast, keratin 14 (*KRT14*) was identified to be downregulated (Fig. 7f).

**Fig. 8:**
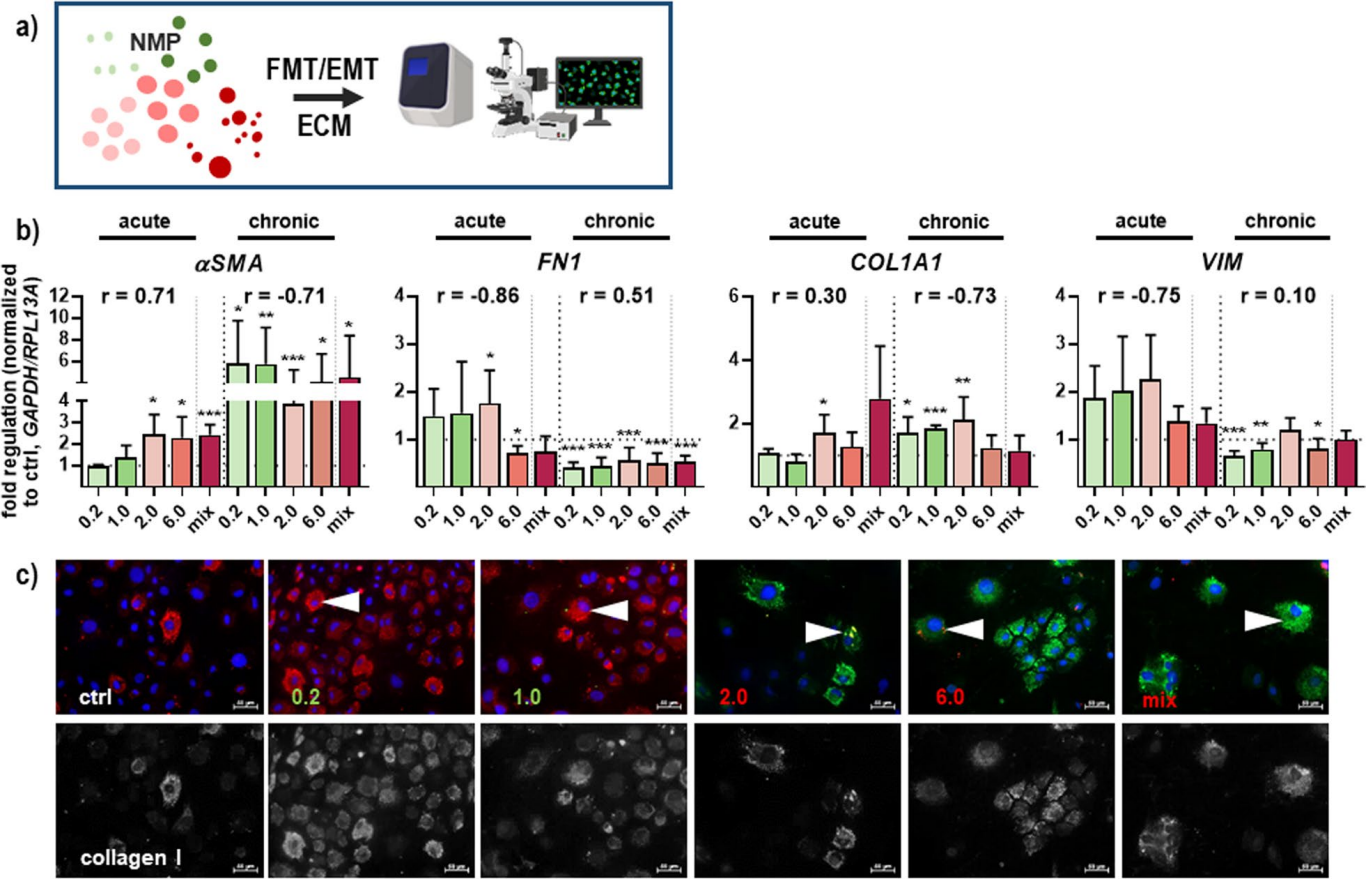
Effects on fibroblast-to-myofibroblast transition (FMT) and extracellular matrix (ECM) targets following NMP uptake in skin cells. **a** Study scheme. **b** qPCR-based gene expression analyses of biomarkers of FMT/EMT such as α smooth muscle actin (*αSMA*) and fibronectin 1 (*FN1)* as well as of ECM component collagen 1A1 (*COL1A1*) and vimentin (*VIM).* Data were normalized to *GAPDH/RLP13A* and untreated controls (ctrl) and presented as mean + SEM. Statistical analysis was done by unpaired, two-tailed *Student*'s *t* test (n ≥ 3) with *p ≤ 0.05, **p ≤ 0.01, and ***p ≤ 0.001. **c** Subcellular distribution and expression of collagen I was analyzed using immunofluorescence microscopy. The cell nuclei were counterstained with DAPI (blue), and strong collagen I-positive cells were marked with arrowheads. Scale bars are 50 µm

To analyze the potential threats of environmental plastic pollutants to transdifferentiation of quiescent fibroblast-to-myofibroblast (FMT) and epithelial-to-mesenchymal phenotype (EMT) as well as to a deposition of ECM proteins (Fig. 8a), we quantified the expression level and/or distribution of α smooth muscle actin (αSMA), fibronectin (FN) 1, collagen (COL) 1A1, and vimentin (VIM). mRNA expression levels of *αSMA* and *COL1A1* were significantly increased after single and continuous exposure with NMP (Fig. 8b), hinting at a strong FTM transition with the differentiation of active fibroblasts into myofibroblasts. Additionally, this apparent increase in expression and cellular deposition was confirmed using fluorescence microscopy, especially in 0.2 µm NMP-treated skin cells. The fluorescence signal and collagen I deposition pattern increased significantly from acute to chronic treatment with NMP but not in untreated controls (Fig. 8c). While the multidomain protein FN1, with its ability to bind simultaneously to many FN molecules such as cell surface receptors and collagen [38], and the intermediate filament protein vimentin (VIM) tended to be upregulated after single NMP exposure, we observed a prolonged down-regulation of pericellular FN matrix and VIM filaments, as shown by their mRNA quantification (Fig. 8b).

**Fig. 9:**
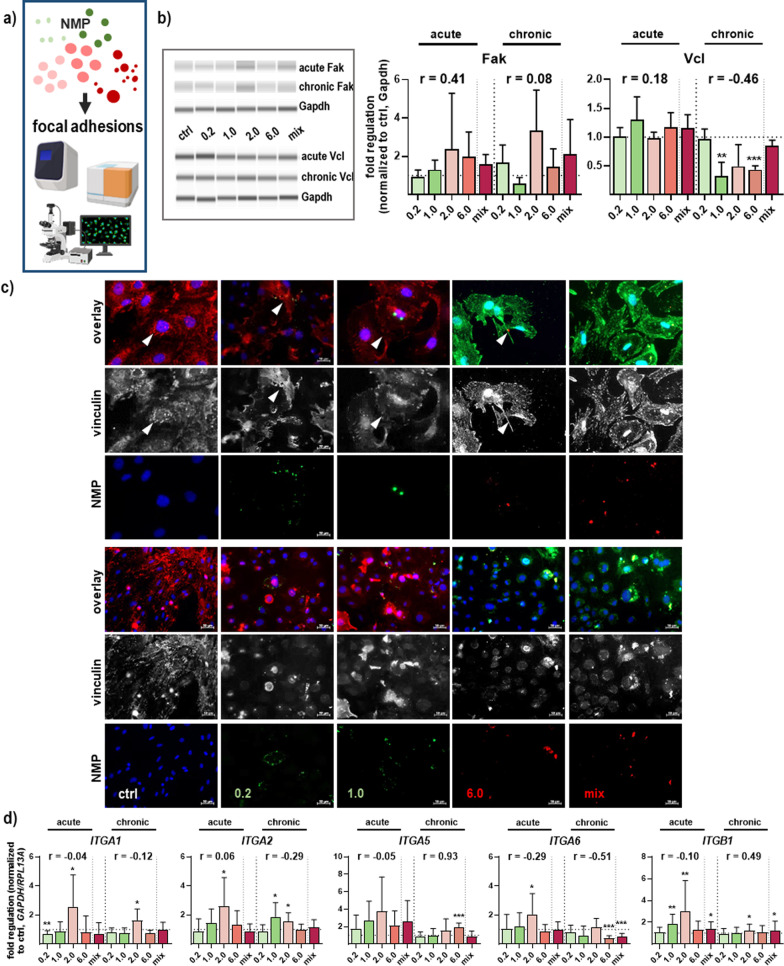
Effects of NMP acting on structural and signaling proteins of integrin adhesion complexes. **a** Study scheme. **b** Representative WES images and quantification of protein expression of focal adhesion kinase (Fak) and vinculin (Vcl) in skin cells. **c** Skin cells were grown on glass coverslips, incubated with NMP, fixed, and subjected to fluorescent labeling of vinculin after acute (top) and chronic exposure (bottom); arrowheads show vinculin-positive cellular protrusions. The cell nuclei were stained with DAPI (blue). Scale bars are 50 µm. **d** mRNA expression levels of several integrins (*ITGA1/2/5/6, ITGB1*) after single and repeated NMP exposure. For qPCR and WES, data were normalized either to *GAPDH/RLP13A* or Gapdh, respectively, and untreated controls (ctrl). Results were presented as mean + SEM. Statistical analysis was done by unpaired, two-tailed Student’s *t* test (n > 3) with *p ≤ 0.05, **p ≤ 0.01, and ***p ≤ 0.001

In the end, the impact of NMP on the adhesive integrin complexes and associated molecules such as focal adhesions was molecularly characterized and quantified employing WES, fluorescence microscopy, and qPCR (Fig. 9a). The molecular basis of focal adhesion is regulated by focal adhesion kinase (Fak) and vinculin (Vcl), showing a slight increase in protein levels of Fak but a significant decrease in Vcl after chronic NMP exposure (Fig. 9b). The structural adaptor protein Vcl, interconnecting signals in focal adhesions, was stronger stained at the leading-edge lamellipodia and filopodia in untreated cells in comparison to NMP-treated cells with smaller sizes of 0.2 and 1 µm after single (arrowheads, top rows Fig. 9c) and prolonged NMP exposure (bottom rows Fig. 9c). Additionally, we found that the mRNA levels of several integrins, such as *ITGA1/2/5/6* and *ITGB1,* were significantly differentially expressed between experimental groups compared to untreated control. However, the expression of integrin *ITGB1* was increased (Fig. 9d).

## Discussion

The presence of plastic in the environment, such as water or food, and the subsequent enrichment of nano- and microplastic particles (NMP) in the body is a potential health threat. Among the different entry routes, humans are exposed to NMP via the skin. However, the consequences of acute or chronic NMP exposure in skin cells are unknown. This is particularly important for chronic NMP exposure, as present in our environment. To this end, this study's major aim was to determine several biological responses, such as cellular uptake, accumulation, and biological effects of NMP in primary skin cells after both single-dose (acute) and repetitive (chronic, long-term) exposure, respectively.

In several previous studies, the plastic particles were relatively large (at 0.5–1 mm) [39] compared to the mammalian cell size of about 1–30 µm. A crucial question for investigating biological consequences is the NMP size, which was evaluated here in the range of 0.2 to 6 µm. However, dermal exposure to NMP bigger than 1 µm is limited by the *stratum corneum* (SC) layer of the skin [40], the preferred size range of NMP observed for a deeper penetration across an intact skin barrier [41]. Although healthy skin is a suitable barrier to prevent NMP penetration, studies on dermal uptake of ambient NMP by damaged skin are, however, relatively limited, variable, and inconclusive. For example, skin barrier dysfunction, particularly damage to the SC layer, is the initial step in developing atopic dermatitis [42]. In addition, plastic surface modifications may facilitate the uptake of particles larger than 1 µm through the SC. Therefore, we find our study setup using primary skin cells suitable as a beneficial proof-of-principle model to determine and characterize NMP-mediated biological consequences in these cells to better understand associated health risks for humans. In vitro*,* a continuous penetration and enrichment of topically applied nano-sized particles for skin tissue and epidermal cells were also shown [43–45]. Generally, several studies showed that small insoluble micro- and nano-sized particles are more likely to accumulate intracellularly [39, 46, 47], corroborating our results of cellular uptake of up to 6 µm NMP in primary skin cells. Furthermore, it has been recognized that polymers usually do not induce notable inflammatory responses if they are used as drug delivery carriers for medications [48]. However, inflammatory responses may occur as a consequence of permanent exposure to and accumulation of NMP and their interference with expression levels of relevant cytokines. In model organisms like zebrafish, cytokine pattern changes have been observed with differential effects of smaller vs. larger particles between 0.1 µm, 5 µm, and 200 µm [49–52]. Our data partially supported this notion as we have seen mainly changes in secretory response after NMP uptake below 6 µm. The upregulation of the tumor necrosis factor-alpha (TNFα), an immune mediator for cell migration, adhesion, apoptosis, and angiogenesis [53], served as an indicator of local inflammatory events [54]. NMP exposure caused a significant increase in TNFα and interleukin 1β (IL1β) secretion, another mediator of an inflammatory response, indicating an NMP size-independent response in skin cells. Interleukin 6 (IL6) acts as a pro-inflammatory cytokine and anti-inflammatory myokine and was also produced in immune cells cultured in the presence of plastic particles smaller than 3 µm [3]. Our data are further consistent with previous studies, where an increase in the secretion of TNFα by treatment with polystyrene particles less than 1 µm in diameter at a concentration of 500 µg/mL was observed, together with a change in the IL6 secretion following treatment with polystyrene particles less than 10 µm [55, 56].

A potential problem with NMP uptake is the possibility of toxic effects by elevated intracellular reactive oxygen species (ROS) levels, as shown for NMP exposure in marine organisms and human epithelial cells [57, 58]. Mice exposed to a single dose of NMP mixtures with different sizes by oral gavage demonstrated increased biodistribution in organs and ROS generation, while pre-treatment with antioxidants reversed the effects [59]. Here, we also suggest that the cellular NMP uptake via skin cells affected the intracellular generation of ROS. Additionally, the glutathione S-transferase (GST) was upregulated NMP-size dependently, hinting at an increase in the detoxification systems caused by the intrinsic formation of ROS and products of oxidative stress by conjugating with glutathione. In this regard, the alpha class of GST exhibits glutathione peroxidase activity, thereby protecting the cells from ROS and peroxidation products. After chronic NMP exposure, we observed a slightly lower toxic effect of NMP, suggesting a cellular adaptation to oxidative stress. Oxidative stress adaptation or hormesis is an important mechanism by which cells respond to environmental and physiological shifts in the level of oxidative stress [60]. Our studies also demonstrated an adaptation of ROS-derived oxidative stress following medical gas plasma exposure in keratinocytes [61]. Quantifying changes in redox conditions in skin cells showed that NMP exposure increased cellular thiol content.

Generally, data on NMP-induced apoptotic responses in primary mammalian cells are scarce. However, in human alveolar epithelial cells [62], NMP-induced generation of ROS and oxidative stress can lead to apoptotic responses [58]. In human colon carcinoma cells [63] and THP1 cells [64], polystyrene NMP also reduced cell viability, corroborating our results after acute but not chronic exposure. The observed decrease of the hypoxia-inducible factor 1α (*HIF1A*) expression after single incubation has reversed with increasing duration of NMP exposure, indicating a regulation through a redox-sensitive mechanism [65]. On the cellular level, apoptosis and mitochondrial dysfunction are consequences of NMP exposure [66]. The B-cell lymphoma 2 (Bcl-2) protein family has essential roles in the regulation of intrinsic, mitochondrial apoptotic cell pathways by either inhibiting (anti-apoptotic) or inducing (pro-apoptotic) apoptosis [33]. In line with this, Bcl-2 protein was less expressed in NMP-treated over untreated skin cells. Bcl-2 expression has been associated with increased resistance of skin cells to DNA damage-induced cell death [34]. As highlighted by immunofluorescence staining, we observed a slight enrichment of phosphorylated histone 2A complex (γH2AX) in the cell nucleus after NMP exposure. Increased γH2AX expression is associated with several biological processes, such as cell proliferation, mitochondrial activity, oxidative stress, and apoptosis, along with its original identification as DNA-damage biomarker in radiobiology [67]. Accumulation of irreparable double-strand breaks (DSB) in fibroblasts, induced by increased intracellular ROS, was also found in aged patients [68, 69]. Albeit this is not directly linked, it appears plausible that NMP-mediated oxidative stress, DNA damage response, and imbalance of apoptotic events may contribute to age-related disorders. Taken together, our results suggest that primary murine skin cells produced intracellular ROS after NMP` uptake, leading to modest cytotoxic responses and changes in chemokine and cytokine secretion profiles.

Exposure to polystyrene NMP causes ROS generation and, subsequently, oxidative stress in vitro [70], affecting the response to oxidative stress pathways in human cells [71]. In mammals, primary body defense mechanisms include the uptake of NMP dependent on particle size through epithelial cells after direct contact [21], and delivery into the digestive tract and intestinal epithelial cells via the blood circulatory system [72, 73]. In response to NMP exposure, several redox-sensitive signaling pathways [58], transcription factors with their downstream targets [74], and adaptive stress response pathways are activated [75]. Physiologically, the expression of nuclear factor-E2-related transcription factor 2 (Nrf2) activates cellular rescue pathways against oxidative injury, inflammation, and apoptotic events [76]. It was observed that the treatment of fetal hepatocytes with fluorescently-labeled polystyrene NMP activated Nrf2 and the Kelch-like ECH-associated protein 1 (Keap1) [74]. An upregulation of superoxide dismutase (SOD) 1 has also been documented in microplastic-treated cells [77, 78]. Levels of catalase (*CAT)* and glutathione peroxidase (GPX) 2 were diminished in the NMP-treated skin cells in a time-dependent manner, which was similar in marine organisms [79, 80]. It appears that mitogen-activated protein kinase (MAPK) signal pathway activation by NMP, through detectable initiation of intracellular ROS production, can be downregulated by a simultaneous increase of antioxidant-responsive element (ARE)-mediated gene expression via Nrf2-dependent mechanisms [81]. In response to acute NMP exposure, the effects in that study were rescued by molecules with antioxidant properties. In contrast, the Nrf2 signaling pathway was negatively affected after prolonged NMP exposure, where some detoxification enzymes were no longer able to neutralize oxidative stress. Hence, the exposure to NMP induced alterations of the anti-oxidant defense mechanism in primary murine skin cells by translocation of Nrf2 into the nucleus, and modulated down-stream expression of oxidative stress-related target genes. Those targets seem to be sensitive markers of oxidative damages following exposure with NMP. These adverse NMP effects may arise from oxidative stress via the intrinsic generation of ROS that acts as a second messenger in signaling.

The dual-function protein β-catenin is involved in regulating cellular adhesion of E-cadherin complex and in gene transcription, where it acts as an intracellular signal transducer in the wingless (Wnt) signaling pathway [82]. In rodent models, increased apoptosis and oxidative stress in the heart was associated with the activation of the Wnt/β-catenin signaling pathway that is also related to myocardium fibrosis [73]. Moreover, hyperplastic wounds and fibroproliferative tumors are often associated with elevated β-catenin protein levels [35, 83, 84]. In the presence of Wnt ligands, β-catenin is not ubiquitinated but accumulates in the nucleus, where it acts as a coactivator for transcription factors of the TCF/LEF family. We found a strong increase of β-catenin expression and a change in the subcellular β-catenin location from membraneous to cytoplasmic and nuclear expression over the four weeks of continuous plastic exposure. Overall, nuclear β-catenin staining is strongly associated with poor prognosis in tumor patients [82, 85]. The disruption of E-cadherin adhesion and its down-regulation, as we have shown after repeated NMP exposure, have essential roles in cell proliferation and are a common epithelial cancer feature [86]. Also, the mRNA level of glycogen synthase kinase (GSK) 3β and the nuclear lymphoid enhancer-biding factor (LEF) 1 were consistent with findings of previous studies. In the absence of Wnt ligands, GSK3β promotes the phosphorylation of β-catenin at key Ser/Thr residues, targeting it for degradation through the ubiquitin-ligase pathway [35]. In addition, NMP administration can interfere with lipid metabolism in the liver and disrupt the thyroid endocrine system [87]. Our data were in accordance with studies where a decreased peroxisome proliferator-activated receptor (PPAR) α and γ mRNA expression in maternal mice and their liver was observed in groups exposed to 5 µm NMP alone (100–1000 µg/mL) [21, 88]. Additionally, PPARα is thought to have anti-inflammatory effects, and its downregulation resulted in fibrosis [89]. The matrix metalloproteinase (MMP) 7 is upregulated in response to β-catenin-mediated LEF1-dependent transcription in NMP-treated skin cells, which was also shown in mesenchymal cells [90]. Our results suggest the possibility that the cellular NMP uptake is related to a dysregulation of β-catenin-mediated downstream signaling and transcription.

One important role of the actin cytoskeleton is regulating the β-catenin / E-cadherin complex between adherent cells. The actin cytoskeleton maintains and changes the cellular shape and structural stability of cells and is involved in cell–cell and cell–matrix adhesion [86]. Individual components of the actin cytoskeleton (e.g., stress fibers, filipodia, lamellipodia, focal adhesions) define the mechanical properties of the cell surface [37], which were strongly changed upon NMP treatment. In addition, multiple diseases include dynamic alterations in the actin cytoskeleton, junctional complexes, and the regulation of substance exchange (e.g., water, ions, and organic molecules) across tissue compartments [91]. Bundles of actin filaments at the cell–matrix contact points are anchored to the integrin family's transmembrane receptors through a multi-molecular complex of junctional plaque proteins [92], which were mainly upregulated as shown for claudin 1 following NMP uptake. However, expression changes of claudin 1 may be a critical risk factor for the pathogenesis and progression of skin diseases such as atopic dermatitis [93]. Additionally, gap junctions, as channels for intercellular cell–cell communication (GJIC), are mainly formed by connexin (Cx) 43, influencing cell–cell contact arrangements and cytoskeletal dynamics, junction assembly, cell polarity, and transcriptional regulation [28]. Several studies point to a critical role of Cx43 in the proper formation and alignment of the actin-based contractile machinery underlying barrier function control [94, 95]. Here, Cx43 was upregulated, indicating a general effect of NMP on GJIC. Overall, our data suggest that cell stressors, such as NMP, are associated not only with inflammatory mediator release but also with reduced cell–cell-contacts, disrupted actin balance, and dysregulated barrier function in primary murine skin cells.

Resident fibroblasts proliferate reactively in response to the microenvironment's external stimulation by secretion of excessive extracellular matrix (ECM) components like collagens. An excessive or prolonged collagen synthesis leads to abnormal scar formation in the skin [96], fibrotic disorders in cardiovascular and pulmonary diseases [97], and is associated with poor prognosis in many cancers [98]. The increase of collagen expression level in response to NMP showed a type I collagen proliferation with fibrosis events and excessive deposition, which was also demonstrated in vivo in aged human-derived cells [50], and in ovaries [99]. Our data also suggest that oxidative stress in NMP-treated primary murine skin cells affected fibronectin fibrillogenesis. Moreover, fibrosis is triggered through β-catenin pathway activation, showing enhanced production of FMT markers like α smooth muscle actin (αSMA). αSMA was significantly increased after single and continuous exposure with NMP, hinting to a strong FTM transition with the differentiation of active fibroblasts into myofibroblasts, which is also linked to clinicopathological characteristics in humans [100]. Several factors of fibrotic processes are attenuated by de novo formation of αSma fibers [101] and mechanistically associated with alterations in the structural integrity of dermal skin layers [102]. Fibrinogen and vimentin changes were observed as indicative of a modulated cellular flexibility and adhesive phenotype of focal adhesions [103, 104]. The structural adaptor protein vinculin, interconnecting signals in focal adhesions, regulating integrin dynamics [105] and actin network [106], was stronger stained at the leading-edge lamellipodia and filopodia in untreated cells in comparison to NMP-treated cells. As structural elements act as transmembrane adhesion receptors, integrins are essential for the interconnection of skin cells and the adhesion to the extracellular matrix (ECM). Integrins are strongly involved in cell movements and migration [107]. The expression levels of integrins were concomitant with a decrease of and disruption in vinculin-associated focal adhesions, which can be signs of cancer and other diseases [103, 108]. It is well-known that adhesive integrin complexes, which selectively allow the penetration of specific molecules, alter gene expression under unusual adhesion processes occur.

There are many different subgroups of keratins, representing a critical intermediary filament in keratinocytes. Krt1 is crucial for maintaining skin integrity and participates in an inflammatory network in murine keratinocytes suggesting a functional link between Krt1 and human inflammatory diseases [109]. Its expression is also significantly correlated with the clinical characteristics of patients with malignant melanoma [110]. Additionally, when tumor cells undergo EMT, the expression of differentiation-specific keratins, such as Krt14, is downregulated, as found in models of cervical melanoma. Thus, oxidative stress in NMP-treated skin cells not only affected the reorganization of the actin cytoskeleton, a prerequisite for changes in cell shape, motility, and gene expression, but also Krt14 downregulation that correlates with more aggressive lesions [111], indicating its clinical significance.

**Fig. 10:**
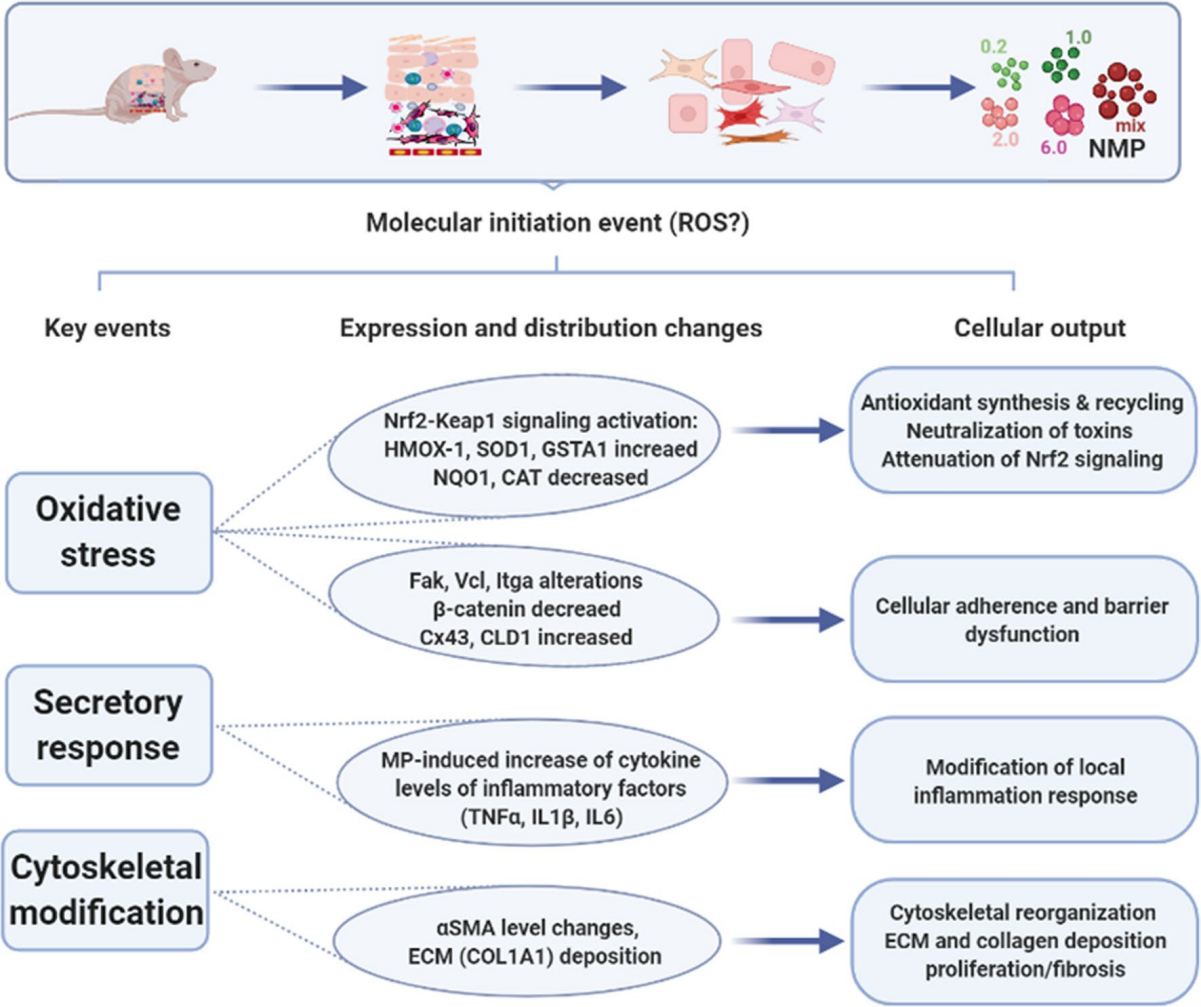
Summary of NMP exposure consequences in murine primary skin cells. Polymeric NMP affected primary cells freshly isolated from murine skin due to their uptake into the cytoplasm, leading to oxidative stress, changes in the secretion profile, and cytoskeletal disruption. Direct cytotoxic effects were less observed

In summary, our results suggested that NMP uptake in primary murine skin cells promotes intracellular ROS production at low cytotoxicity while highlighting mechanistic pathways and modulation of transcriptional patterns of apoptosis-related genes. In addition, NMP treatment modulated the secretory profile. It activated Nrf2-driven antioxidant defense responses, accompanied by altered β-catenin signaling in the skin cells (Fig. 10). Moreover, changes such as alterations of expression and/or distribution of molecules of adhesive integrin complexes, dynamic regulation of structural proteins and cytoskeleton architecture, and collagen fibers underline the ability of NMP to modulate cytoskeletal components with disease relevance in humans. Future studies may investigate such pathways in vivo by using genuine mixtures of environmentally-retrieved plastic particles.

## Supplementary Information

**Additional file 1**: ** Table S1**. Murine gene-specific primer used in qPCR.; **Figure S1**. Expression of HO-1 in skin cells following NMP uptake. Representative images of 0.2 µm (a), 1.0 µm (b), 2.0 µm (c), and 6.0 µm (d) NMP uptake in skin cells in brightfield and fluorescence channels. The cell nuclei were stained with DAPI (blue). Scale bar is 50 µm;** Figure S2**. Expression of HO-1 in skin cells following NMP uptake. Distribution and expression of HO-1 after nuclear Nrf2 translocation and activation were observed after NMP uptake using immunofluorescence labeling in skin cells. The cell nuclei were stained with DAPI (blue). Scale bar is 50 µm;** Figure S3**. Effects on β-catenin signaling in skin cells following NMP uptake. Expression and distribution of β-catenin after chronic exposure to 0.2–6 µm and mix NMP. The cell nuclei were counterstained with DAPI (blue). Scale bar is 50 µm.

## Data Availability

The datasets supporting the conclusions of this article are included within the article and can be retrieved from the corresponding author upon reasonable request.
