# Supplementary material for: Short- and long-term polystyrene nano- and microplastic exposure promotes oxidative stress and divergently affects skin cell architecture and Wnt/beta-catenin signaling
Source: Part Fibre Toxicol. 2023 Jan 16;20:3. doi: 10.1186/s12989-023-00513-1 (PMC9844005; doi:10.1186/s12989-023-00513-1)

Supplemental Data

**Table S1**

| **Gene name** | **Gene ID** | **Primer sequences (3`- 5`)** |
| --- | --- | --- |
| nuclear factor erythroid 2-related factor 2 | *NRF2* | GAG TCG CTT GCC CTG GAT ATC  TCA TGG CTG CCT CCA GAG AA |
| heme oxygenase 1 | *HMOX1* | TGA AGC AGG CAT CTG AGG G  CGA AGG TGG AAG AGT GGG AG |
| NAD(P)H dehydrogenase[quinone] 1 | *NQO1* | GGC ATC CAG TCC TCC ATC AA  GTT AGT CCC TCG GCC ATT GTT |
| kelch-like ECH-associated protein 1 | *KEAP1* | CGG GGA CGC AGT GAT GTA TG  TGT GTA GCT GAA GGT TCG GTT A |
| catalase | *CAT* | CAG AGA GCG GAT TCC TGA GAG A  CTT TGC CTT GGA GTA TCT GGT GAT |
| superoxide dismutase [Cu-Zn] | *SOD1* | GAA ACA AGA TGA CTT GGG CAA AG  TTA CTG CGC AAT CCC AAT CA |
| glutathione peroxidase 1 | *GPX2* | GTG GCG TCA CTC TGA GGA ACA  CAG TTC TCC TGA TGT CCG AAC TG |
| glutathione-disulfide reductase | *GSR* | TCG GAA TTC ATG CAC GAT CA  GGC TCA CAT AGG CAT CCC TTT |
| tumor necrosis factor-alpha | *TNFa* | TCT CAT GCA CCA CCA TCA AGG ACT  ACC ACT CTC CCT TTG CAG AAC TCA |
| interleukin 1β | *IL1β* | GCA ACT GTT CCT GAA CTC AAC T  ATC TTT TGG GGT CCG TCA ACT |
| interleukin 6 | *IL6* | ATC CAG TTG CCT TCT TGG GAC TGA  TAA GCC TCC GAC TTG TGA AGT GGT |
| β actin | *ACTB* | TTG CTG ACA GGA TGC AGA AG  ACA TCT GCT GGA AGG TGG AC |
| smooth muscle actin α | *αSMA* | CCC AGA CAT CAG GGA GTA ATG G  TCT ATC GGA TAC TTC AGC GTC A |
| collagen 1A1 | *COL1A1* | GCT CCT CTT AGG GGC CAC T  ATT GGG GAC CCT TAG GCC AT |
| gap junction protein 1 | *CX43* | TTG GCT CAC GTG TTC TAT GT  ACC TCTCAT CTT CAC CTT GC |
| heat shock protein 70 | *HSP70* | GGC TGA CAA GAA GAA GGT GC  CTG GTA CAG CCC ACT GAT GA |
| heat shock protein 90α | *HSP90α* | GAC GCT CTG GAT AAA ATC CGT T  TGG GAA TGA GAT TGA TGT GCA G |
| vinculin | *VCL* | GCT TCAGTC AGACCC ATA CTC G  AGG TAA GCA GTA GGT CAG ATG T |
| focal adhesion kinase | *FAK/PTK2* | GAG TAC GTC CCT ATG GTG AAG G  CTC GAT CTC TCG ATG AGT GCT |
| integrin A1 | *ITGA1* | GAC AGC CCT TGG AAT AGA CAC  GTT GTC ATG CGA TTC TCC ATC A |
| integrin A2 | *ITGA2* | TGT CTG GCG TAT AAT GTT GGC  TGC TGT ACT GAA TAC CCA AAC TG |
| integrin A5 | *ITGA5* | TGC AGT GGT TCG GAG CAA C  TTT TCT GTG CGC CAG CTA TAC |
| integrin A6 | *ITGA6* | GGG ATC GTC CGT GTA GAA CAA  TCT CTC CAC CAA CTT CAT AGG G |
| integrin B1 | *ITGB1* | TGG TCA GCA ACG CAT ATC TGG  GAT CCA CAA ACC GCA ACC T |
| fibronectin 1 | *FN1* | GCT CAG CAA ATC GTG CAG C  CTA GGT AGG TCC GTT CCC ACT |
| vimentin | *VIM* | CGT CCA CAC GCA CCT ACA G  GGG GGA TGA GGA ATA GAG GCT |
| wingless-type MMTV integration site 1 | *WNT1* | CGACTGATCCGACAGAACCC  CCATTTGCACTCTCGCACA |
| wingless-type MMTV integration site 7a | *WNT7a* | TCAGTTTCAGTTCCGAAATGGC  CCCGACTCCCCACTTTGAG |
| β-catenin 1 | *CTNNB1* | CCC AGT CCT TCA CGC AAG AG  CAT CTA GCG TCT CAG GGA ACA |
| E-cadherin | *CDH1* | CAC CTG GAG AGA GGC CAT GT  TGG GAA ACA TGA GCA GCT CT |
| nuclear factor LEF1 | *LEF1* | GCC ACC GAT GAG ATG ATC CC  TTG ATG TCG GCT AAG TCG CC |
| Jnk for c-jun N-terminal kinase α | *c-JUN* | GTCCTCCATAAATGCCTGTTCC  GATGCAACCCACTGACCAGAT |
| peroxisome proliferator activator receptor delta | *PPARD* | TCCATCGTCAACAAAGACGGG  ACTTGGGCTCAATGATGTCAC |
| matrix metalloproteinase 7 | *MNMP7* | CTTACCTCGGATCGTAGTGGA  CCCCAACTAACCCTCTTGAAGT |
| claudin 1 | *CLD1* | GGG GAC AAC ATC GTG ACC G  AGG AGT CGA AGA CTT TGC ACT |
| vascular endothelial factor | *VEGF* | AAC GAT GAA GCC CTG GAG TG  GAC AAA CAA ATG CTT TCT CCG |
| glycogen synthase kinase 3 α | *GSK3β* | AAG CTC TGC GAT TTT GGC AGT  GAG TTC TGG AGC ACG GTA GTA |
| glyceraldehyde 3-phosphate dehydrogenase | *GAPDH* | CAT GGC CTC CAA GGA GTA AG  TGT GAG GGA GAT GCT CAG TG |
| ribosomal protein 13A | *RLP13A* | AGC CTA CCA GAA AGT TTG CTT AC  GCT TCT TCT TCC GAT AGT GCA TC |

**Figure S1**


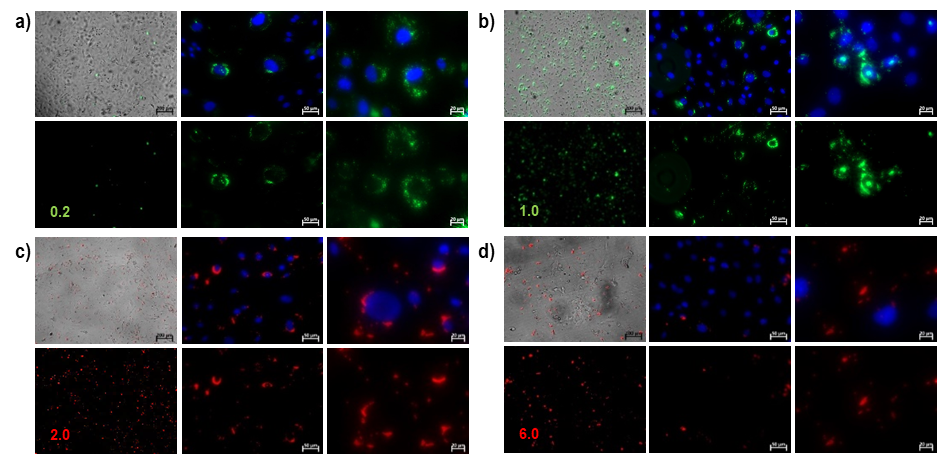


**Figure S2**


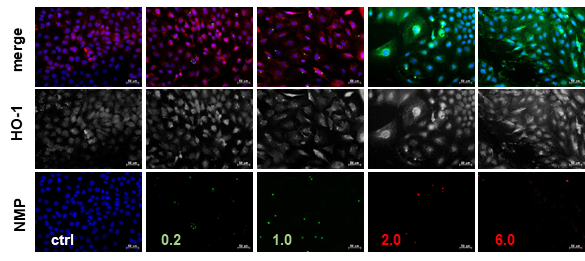


**Figure S3**


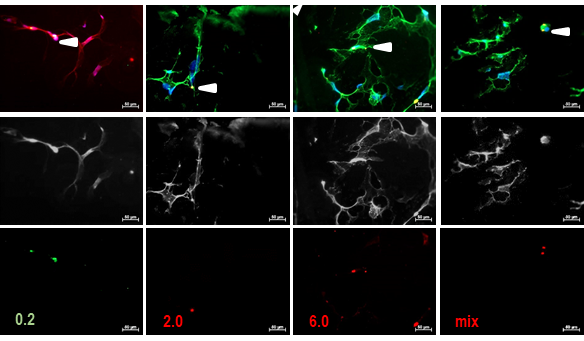

Supplement: Supplementary file 1 — Additional file 1: Table S1. Murine gene-specific primer used in qPCR.; Figure S1. Expression of HO-1 in skin cells following NMP uptake. Representative images of 0.2 µm (a), 1.0 µm (b), 2.0 µm (c), and 6.0 µm (d) NMP uptake in skin cells in brightfield and fluorescence channels. The cell nuclei were stained with DAPI (blue). Scale bar is 50 µm; Figure S2. Expression of HO-1 in skin cells following NMP uptake. Distribution and expression of HO-1 after nuclear Nrf2 translocation and activation were observed after NMP uptake using immunofluorescence labeling in skin cells. The cell nuclei were stained with DAPI (blue). Scale bar is 50 µm; Figure S3. Effects on β-catenin signaling in skin cells following NMP uptake. Expression and distribution of β-catenin after chronic exposure to 0.2–6 µm and mix NMP. The cell nuclei were counterstained with DAPI (blue). Scale bar is 50 µm. [file 12989_2023_513_MOESM1_ESM.docx]
